# Feature Selection Methods for Identifying Genetic Determinants of Host Species in RNA Viruses

**DOI:** 10.1371/journal.pcbi.1003254

**Published:** 2013-10-10

**Authors:** Ricardo Aguas, Neil M. Ferguson

**Affiliations:** MRC Centre for Outbreak Analysis and Modelling, Department of Infectious Disease Epidemiology, Imperial College London, Faculty of Medicine, London, United Kingdom; University of California San Diego, United States of America

## Abstract

Despite environmental, social and ecological dependencies, emergence of zoonotic viruses in human populations is clearly also affected by genetic factors which determine cross-species transmission potential. RNA viruses pose an interesting case study given their mutation rates are orders of magnitude higher than any other pathogen – as reflected by the recent emergence of SARS and Influenza for example. Here, we show how feature selection techniques can be used to reliably classify viral sequences by host species, and to identify the crucial minority of host-specific sites in pathogen genomic data. The variability in alleles at those sites can be translated into prediction probabilities that a particular pathogen isolate is adapted to a given host. We illustrate the power of these methods by: 1) identifying the sites explaining SARS coronavirus differences between human, bat and palm civet samples; 2) showing how cross species jumps of rabies virus among bat populations can be readily identified; and 3) *de novo* identification of likely functional influenza host discriminant markers.

## Author Summary

Moving away from genome scan methods used for human GWAS (ultimately inappropriate for the short highly polymorphic genomes of RNA viruses), our work shows the power and potential of multi-class machine learning algorithms in inferring the functional genetic changes associated with phenotypic change (e.g. crossing a species barrier). We show that even distantly related viruses within a viral family share highly conserved genetic signatures of host specificity; reinforce how fitness landscapes of host adaptation are shaped by host phylogeny; and highlight the evolutionary trajectories of RNA viruses in rapid expansion and under great evolutionary pressure. We do so by (for each dataset) unveiling a set of phenotype characteristic mutations which are shown to be functionally relevant, thus providing new insights into phenotypic relationships between RNA viruses. These methods also provide a solid statistical framework with which the degree of host adaptation can be inferred, thus serving as a valuable tool for studying host transition events with particular relevance for emerging infectious diseases. These methods can then serve as rigorous tools of emergence potential assessment, specifically in scenarios where rapid host classification of newly emerging viruses can be more important than identifying putative functional sites.

## Introduction

Emerging infectious diseases (EIDs) continue to represent a significant public health threat, as illustrated by the 2009 H1N1 influenza pandemic and the 2003 severe acute respiratory syndrome (SARS) epidemic. Of particular concern are the 60%+ of EIDs of zoonotic origin [1], [2]. In addition to influenza and SARS [3], notable examples include hantaviruses [4], Nipah and Hendra viruses [5] and HIV [6].

While predicting the emergence of new pathogens is likely to remain an unachievable goal for the immediate future, an emphasis of current research has been to try to identify ecological, behavioural and biological predictors of cross-species transmission and consequent disease emergence [2], [7], [8], [9], [10]. The wealth of pathogen sequence data becoming available makes identification of pathogen genomic markers of emergence one of the more promising approaches [11], particularly for RNA viruses given their high mutation rate and resulting high diversity at the population level [12].

The identification of genetic markers predicting cross-species disease emergence faces many of the same challenges as genotype-to-phenotype mapping in other spheres, such as human genome-wide association studies of risk factors for chronic diseases [13]. Principle among these are relatively small sample sizes coupled with a very large number of potential explanatory variables (single nucleotide substitutions and their interactions) [14], [15]. However, the much higher frequency of polymorphisms in RNA viruses and their fast population-level evolution offers unique challenges and opportunities.

While most viral variants generated in a specific host are selectively neutral in that host, upon crossing the species barrier they are under strong selective pressure. We expect selection to shape the relative frequencies of viral variants found in donor and recipient species. Specific hosts impose specific evolutionary landscapes on viruses which will translate into signature genetic sequences. We therefore expect comparisons of allele frequencies between sequences of the same pathogen isolated from different hosts to reveal a large subset of alleles which are conserved between host species and a smaller subset of host specific alleles. This comparison can be performed by statistical techniques able to discriminate phenotype (host) relevant variables (alleles). Here we apply feature selection methods which identify a subset of variable sites which can be used to build a robust phenotype classifier [16]. We focus on one algorithm for classification - the random forest algorithm (RFA) - that offers excellent performance in classification tasks, providing direct measures of variable importance and classification error [17].

## Results

Our goals are two-fold. First, we investigate how well feature-selection algorithms such as RFA can reliably classify RNA viruses according to their host species reservoir, thereby giving insight into pathogen evolution, and the frequency of cross-species transition events. Identification of functional polymorphisms is not critical in meeting this goal, though clearly is desirable. Second, we evaluate how well RFA can identify sets of sites that are functionally relevant to the phenotype of interest (in this case host species), in the context of dense RNA virus genomes and their high degree of linkage.

**Figure 1:**
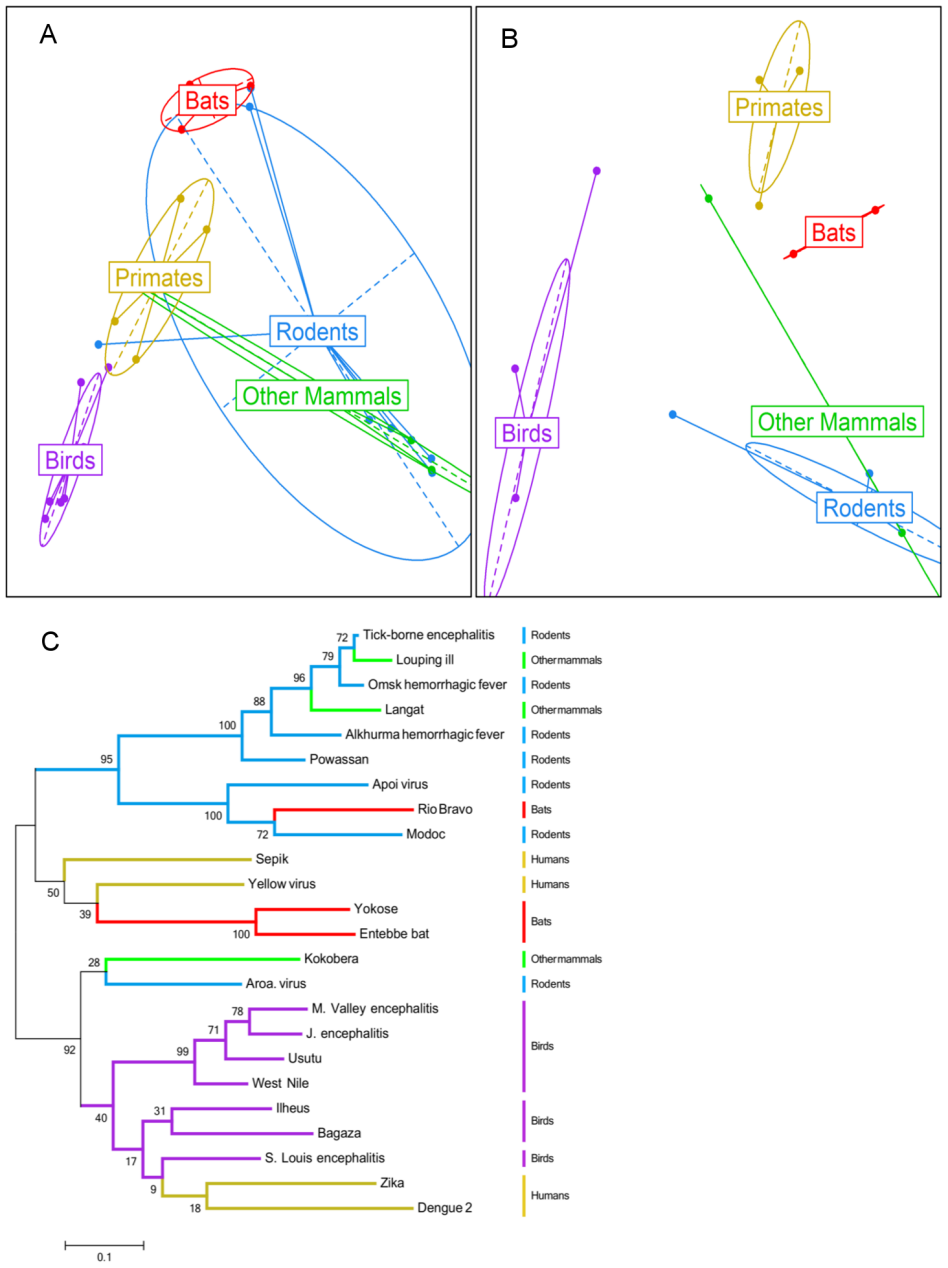
Feature selection of host specific genetic signatures within Flaviviridae. The scatterplots display the first two principal components of the PCA undertaken using allele frequency information from (a) Flaviviruses' full polymerase sequences and (b) an alignment of the amino acids selected by the random forest algorithm. The maximum likelihood phylogenetic tree obtained from full polymerase sequences is presented in (c). Tree branch lengths reflect the number of amino acid differences per sequence.

We first analyse polymerase gene sequences of RNA viruses to identify the genetic signatures predicting host species. As an example, Figure 1a represents the diversity of Flavivirus polymerase amino acid sequences (Table S2). Here we use principal component analysis (PCA) solely to visualise the variation between samples, not as a classification tool. Figure 1b illustrates how feature selection identifies amino acid positions which robustly classify samples by host species, resulting in clustering of samples which infect the same reservoir. The clustering of samples seen in the PCA plot is similar to that seen in the maximum likelihood tree (Figure 1c), supporting the use of PCA as a useful tool for generating low-dimensional representations of genetic variation.

**Figure 2:**
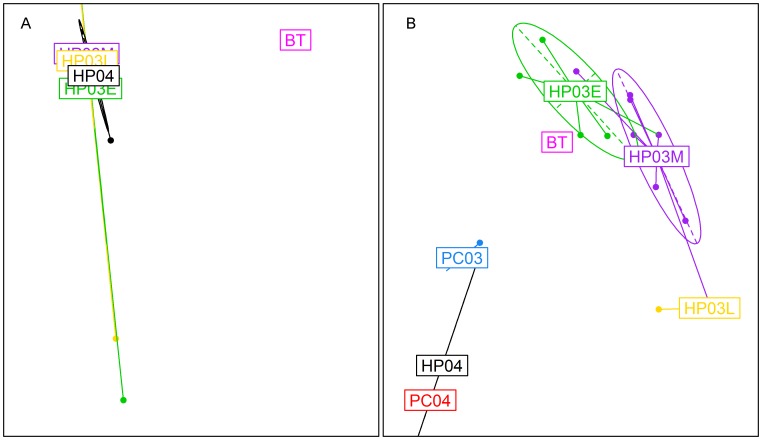
SARS coronavirus species transitions and evolution. The first two principal components of the PCA undertaken using (a) SARS coronavirus complete spike protein nucleotide sequences, and (b) nucleotides selected by the RFA. Viral groups, defined by host species and season, are represented by ellipses of different colours: Human patient samples from 2002/2003 collected in early, mid and late epidemic phase are HP03E (green), HP03M (purple) and HP03L (yellow); 2004 Human samples are labelled HP04 (black); palm civets samples collected in 2003 and 2004 are labelled PC03 (blue) and PC04 (red); bat samples are labelled BT (magenta).

**Figure 3:**
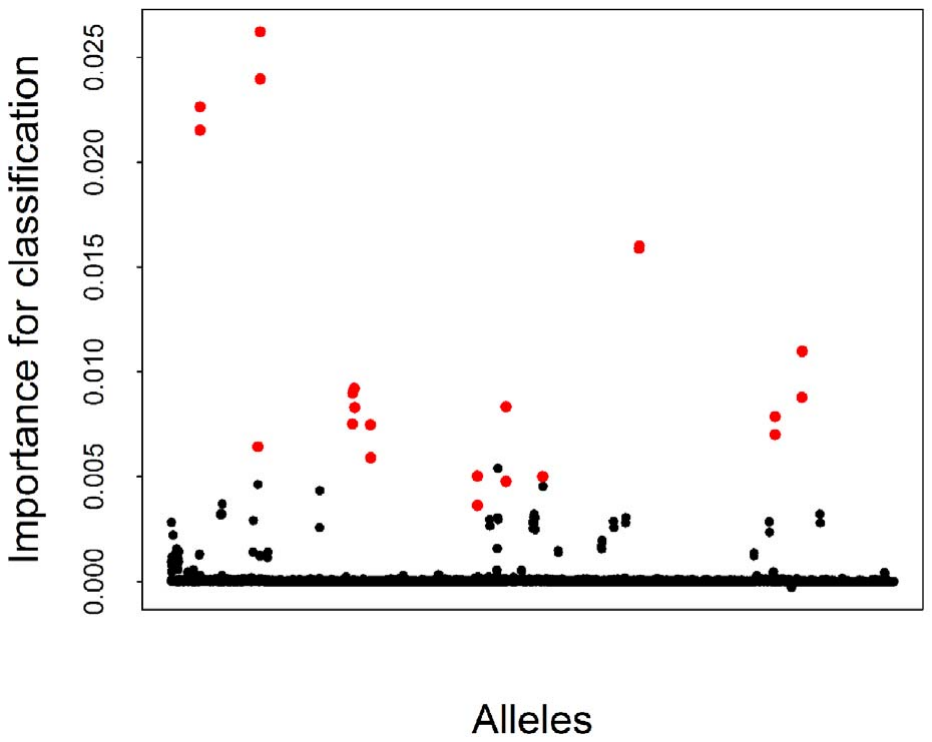
Allele importance for host reservoir classification of SARS-like coronaviruses. The alleles which were identified as significant for classification by the feature selection algorithm are represented by red points.

Second, we examine the potential of RFA applied as a phenotypic classifier to give insight into cross-species disease emergence. In this case, analysis of sequences of viruses which have fully adapted to particular host species – as in the Flavivirus example – is insufficient to distinguish between the subset of mutations required to allow cross-species emergence and later non-essential mutations which further increase viral fitness in a new species. We therefore need to examine data collected from zoonotic outbreaks. The 2003 SARS epidemic is a good example of a zoonosis which rapidly developed a high level of transmissibility in humans [3], [18], [19]. The pathogen was rapidly identified [3] and the origin of the virus was initially traced back to palm civets [19], before bats were identified as the natural reservoirs of SARS-like coronaviruses [20]. We applied the RFA to nucleotide sequences of the spike protein of SARS-like coronaviruses (Table S3), recovered from human patients and palm civets from the 2003 and 2004 epidemics and bat sequences available in the Genbank database. Figure 2a illustrates the extent to which bat sequences differ from the human and palm civet sequences recovered in China in 2002–2004, and also highlights the similarity of palm civet and human sequences [19]. Analysis of the variation in the selected host-discriminant viral alleles (highlighted in Figure 3) reveals interesting relationships between host reservoirs (Figure 2b). Firstly, there is noticeable genetic variation in the samples from human SARS patients collected in the early and mid-stages of the 2003 epidemic, compatible with adaptation of the virus to a new host species. The late 2003 samples were less variable, suggesting selective pressures may by then have stabilized [21]. Secondly, human patient samples from a small outbreak in January 2004 are more closely related to palm civet 2004 samples than to any human sample from the previous year, indicating that the 2004 outbreak represented an independent cross species transition [22]. The palm civet samples from 2003 were collected a few months after the human epidemic ended so there might have been an accumulation of mutations responsible for the substantial distance between palm civet 2003 samples and human 2003 samples. However, the close proximity between the bat samples and the first samples from the human 2003 epidemic suggests that the transition from palm civet to human occurred quite rapidly after the transition from bat to palm civet. With respect to our second goal – identifying functional relevant sites – it is notable that 12 of the 15 positions identified by feature selection coded for non-synonymous substitutions (Table S4), most of which are mapped onto the surface of the spike protein. It should be noted that of the 15 positions identified in the current study, 13 overlap with those found in [23]. The functional relevance of the two unique positions (239 and 311) found here and that of the 13 unique positions identified in [23] is not clear. When running the RFA for amino acid sequences of the same viruses, we obtain a subset of 12 significant amino acid positions that are coded for by the exact same non-synonymous substitutions highlighted by the RFA conducted on the nucleotide sequences.

**Figure 4:**
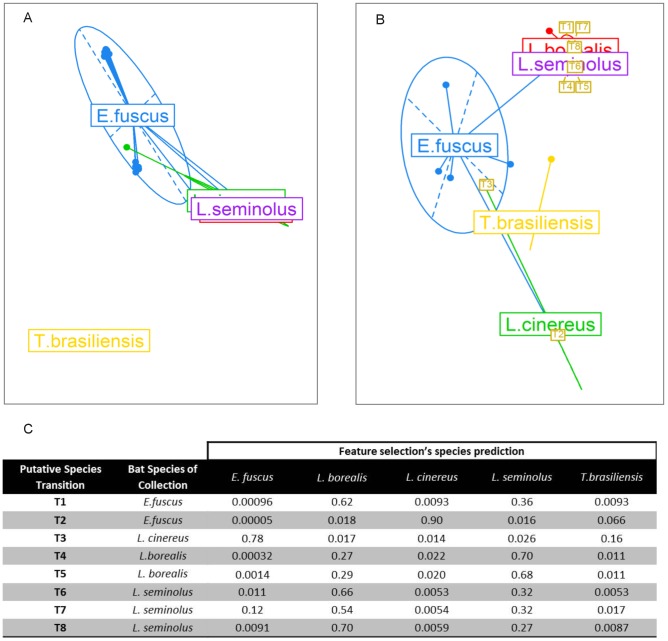
Cross-species transition events of rabies viruses in bats. The first two principal components of the PCA undertaken using (a) complete Rabies virus nucleoprotein sequences, and (b) an alignment of nucleotides selected by the RFA. The ellipses of different colours represent the bat species in which virus samples were collected. Eight putative cross-species transmission events are highlighted in yellow with the respective predicted bat species of origin shown in (c).

High mutation rates in RNA viruses facilitate the overcoming of host specific barriers [24] particularly in ecological settings where hosts display high contact rates [8], [22]. However, cross-species transfer seems to be favoured between closely related host species [9], [25], [26], [27], suggesting that the fitness landscape of host adaptation is shaped by host phylogeny. Streicker and colleagues [26] defined lineages of rabies virus associated to particular bat taxa, identifying 43 cross species transmission events involving 15 bat species. Here we reanalyse the complete nucleoprotein sequences available for five of those bat species (Table S5). PCA applied to these sequences (Figure 4a) shows how viruses collected from 3 of the bats species (*L. borealis, L. seminolus, L. cinereus*) are extremely similar, with a substantially divergent lineage infecting *E. fuscus* bats and an isolated small cluster of viruses seen in *T. brasiliensis*. Applying RFA to predict host species to these sequences allows discrimination of *L. cinereus* specific traits (Figure 4b), but does not significantly separate the *L. borealis* and *L. seminolus* clusters. This suggests that transmission of rabies virus between these two bat species is much more frequent than between any other pair of species examined. The advantage of RFA compared with phylogenetic methods is that it allows a probability of “belonging” to each host bat species to be estimated for each virus sample. Thus we can examine whether a virus isolated in one species is in fact native to a different host species. Figure 4b highlights the 8 outlier sequences (T1–T8) in this dataset – viruses which are closer to rabies viruses native to a different species from that in which they were isolated. For these 8 viruses, Figure 4c gives the RFA classification probabilities of these viruses to the 5 different host species considered. In six cases, the cross-species transitions thus identified agree with those identified in [26]. Five of these 8 transitions occurred between *L. borealis* and *L. seminolus*. This, and the relatively poor ability of RFA to choose between these species in classifying viruses (Table S6), suggests that phylogenetic closeness between host species (Figure S4) facilitates cross-species transmission.

To address our second goal of investigating the functional relevance of identified discriminant features, we applied RFA to a collection of influenza A samples from distinct host species focusing on two viral segments that have been suggested to be major determinants of host range and virulence [28]. First, as a critical validation of the RFA, we analysed H1N1 hemagglutinin (HA) amino acid sequences collected in human (pre and post 2009 pandemic) and swine hosts, since multiple sources of empirical evidence for the functional relevance of specific amino acids in that gene are available [29], [30], [31]. Second, we analysed the PB2 Influenza A gene, since it is highly conserved across subtypes and its evolution has been hypothesised to reflect host specific adaptation [32].

**Figure 5:**
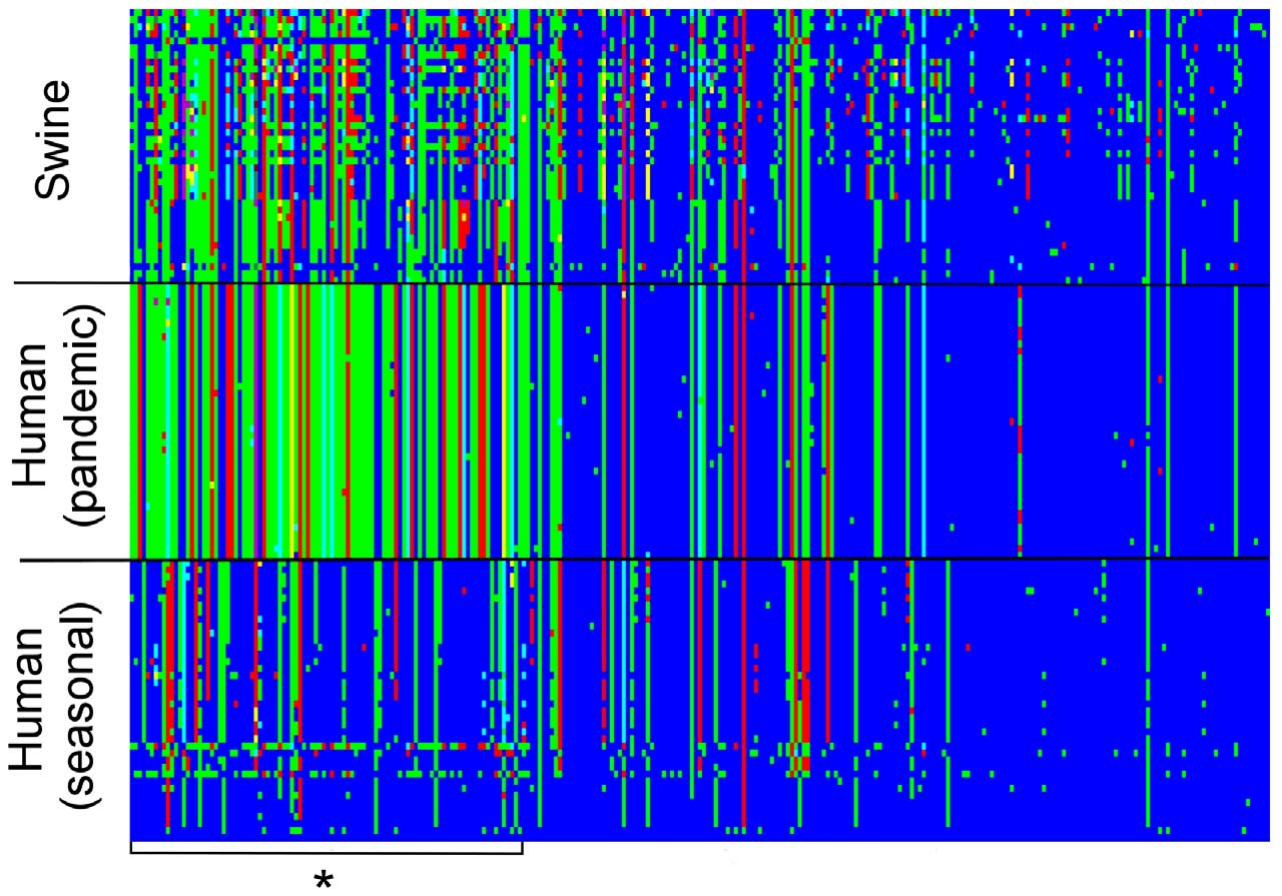
Allele diversity across samples of influenza A H1N1 HA sequences collected from human (pre and post 2009 pandemic) and swine hosts. Each vertical stripe represents allelic variance for a specific amino acid residue in three blocks of 40 sequences (taken at random) per host/virus type. The block of amino acids marked by an asterisk refers to the 100 residues to which the RFA has attributed the highest significance in explaining the allelic differences observed between groups. The ordering of other amino acids follow that of the HA gene. For each position (column) the allele present in the first human (seasonal) virus is colored blue. Moving from bottom to top, different alleles at the same position are then sequentially colored green, red, cyan, yellow and purple. Non polymorphic sites are not shown.

The HA analysis serves not only as an assessment of the functional relevance of the positions being highlighted as host specific by RFA, but also as a benchmark of the method by direct comparison with a recently published study [33] which made use of an alternative feature-selection algorithm (Adaboost). We compare algorithm performance on three levels: prediction ability, percentage of selected amino acids in functionally relevant positions, and overlap of selected amino acids. We use full HA segment amino acid sequences and analyse the proportion of selected amino acids that fall in the Receptor Binding Domain (RBD), and in known antigenic sites. Table 1 summarises our findings by comparison with the Adaboost results [33]. There is substantial overlap with the sets of relevant positions between the two methods, although RFA seems to consistently identify a larger proportion of amino acids in HA's receptor binding domain (RBD), particularly those that are also known antigenic sites, with a greater predictive ability. Even if one were to aggregate the Adaboost results (Adaboost can only undertake binary classification, so two comparisons were needed to explore host-specific determinants for 3 virus groups), that algorithm identifies 47 significant positions, 20 (42.5%) of which belong to the RBD, 7 (35%) in known antigenic sites. A multi-class RFA is able to identify a significant larger subset of amino acids in known antigen sites (12 in the RBD plus 2 others), the functional relevance of which can be explored in future experimental studies. Table S7 lists all the positions selected as significant, while Figure 5 portrays allelic diversity across the HA samples analysed and gives clear intuition into why the identified sites were selected by RFA. We should note the absence of the 190 and 225 mutations (hallmark mutations of human-adapted H1N1 HA) from the subset of significant residues determined by RFA. Although these mutations confer optimal contact with the sialic acid receptors [29], we find that 190D is highly conserved throughout our sequences, contrasting with the 190E amino acid found in avian samples. Residue 225 is picked as one of the 100 most informative sites for host discrimination by the RFA. All the virus groups examined contain samples with the 225D allele, while the 225G allele (the consensus in avian viruses) is present in some seasonal human and swine samples. Had we included avian samples in the analysis, the 225 positions would certainly be classified as highly host discriminant. Here, we identify other mutations which have empirically been found to influence contact with the α2–6 glycans, either by providing additional anchoring sites for the sialic acid (position 145); by forming a network interacting with Asp190 (186, 187 and 189); or by modulating the stability of those contacts (219 and 227) [34], [35]. Identified positions 155 and 131 are also thought to play a relevant role in binding to sialic acid receptors [34], [36].

**Table 1:** RFA selected set of putative functionally relevant host discriminating amino acids in H1N1 influenza HA compared with those found with the Adaboost algorithm.

Viruses	In Receptor binding domain^1^	In known antigenic sites^2^	Selected known antigenic sites	Prediction error
*ADABOOST* 3 *(2 way analysis)*				
Human+Pandemic Human	9/18 (50%)	4/9 (44.4%)	145,206,171,225	0.02
Pandemic Human+Swine	15/34 (44%)	5/15 (33.3%)	225,171,188,206,189	0.1
*RFA (2 way analysis)*				
Human+Pandemic Human	22/39 (56%)	12/22 (54.5%)	81, 145, 156, 158, 159, 163, 169, 171, 187, 189, 196, 198, 208	0.0078
Pandemic Human+Swine	17/30 (56%)	7/17 (41.2%)	80, 132, 140, 145, 149, 171, 188, 208	0.0024
*RFA (3 way analysis)*				
Human+Pandemic human+Swine	26/49 (53%)	12/26 (46.2%)	80, 81, 145,156, 158, 159, 169, 171,187, 188, 189, 196, 198, 208	0.016

1 The receptor binding domain refers to positions 114 through 268 of the HA segment.

2 The antigenic sites considered here are those defined as such in [60].

3 The Adaboost algorithm as implemented in [33].

**Figure 6:**
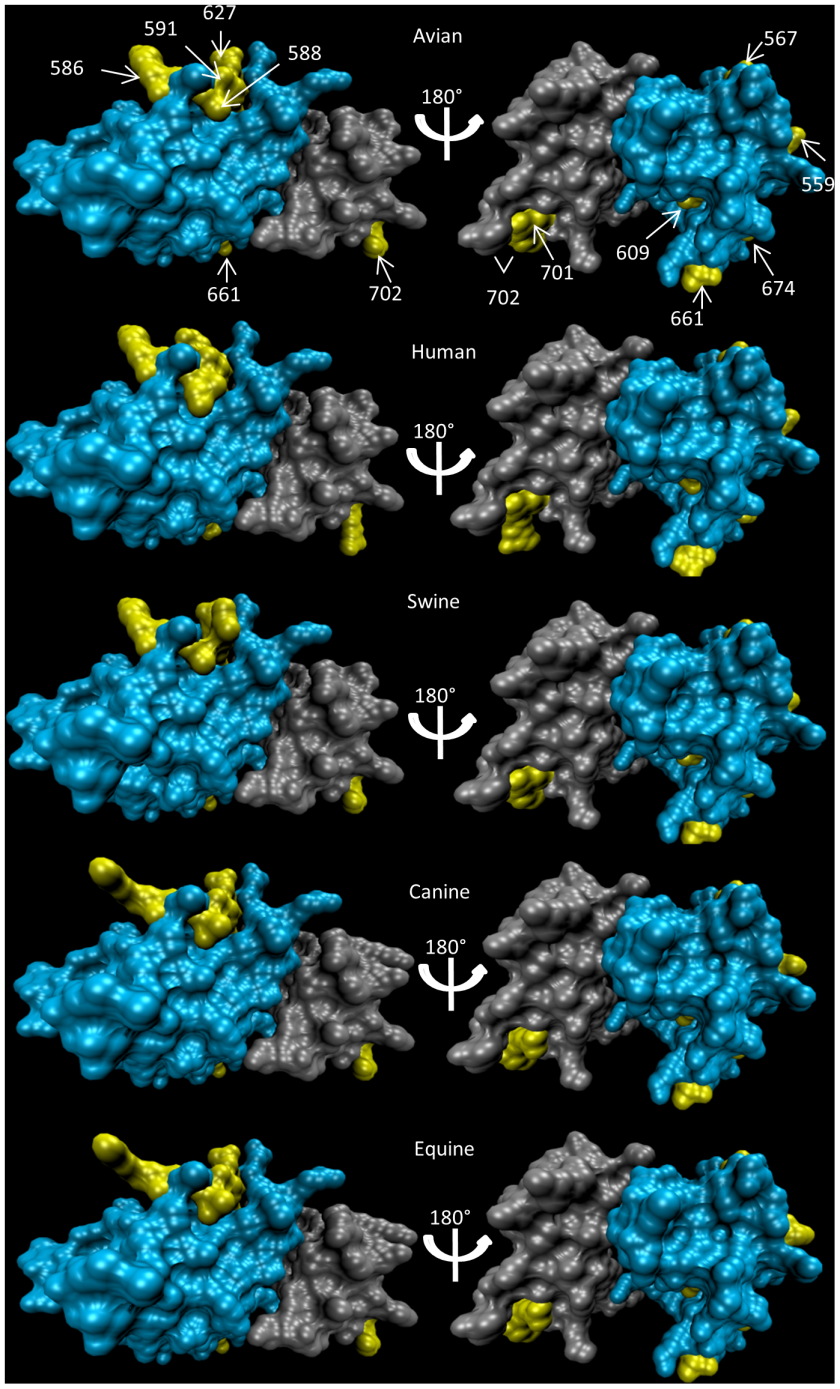
Computationally predicted structure of the 531–738 subset of amino acids in the PB2 subunit of the polymerase protein of influenza A viruses. For structural prediction we used the consensus sequence for the subset of viruses' samples collected from each host species. These sequences contain two functional domains: the 627 (in cyan) and the NLS binding (in grey) domains. Highlighted in yellow are the amino acids which were identified by the RFA as discriminating host species.

Feature selection performed on the PB2 segment highlights subtype transcending functionally relevant amino acids from sequences of 7 influenza subtypes (H1N1, H1N2, H2N2, H3N2, H5N1, H3N8, H7N7), collected in 5 different hosts (humans, birds, pigs, dogs, and horses), as detailed in Table S8. Overall, we identified a subset of 23 host discriminant positions (Table S9), out of which only 7 fall outside known functional domains [37], [38]. Our results are substantially congruent (overlap of 7 identified positions out of 12) with those of a phylogenetic study aimed at identifying amino acid sites with strong support for different selection constraints in human and avian viruses [39], even though our analysis is not limited to differences between these two hosts. A closer look at the identified sites in the most extensively studied functional domains (the 627 and NLS domains) reveals that all lie on the surface of the protein (Figure 6), with mutations at positions 588, 591,627, and 702 being responsible for the most drastic conformational changes. Analysis of the physiochemical properties of the selected amino acids reveals side chain charge reversals in positions 591 and 627 (Table S9). The insertion of a lysine in an otherwise avian adapted H5N1 virus (which is unable to infect humans) has been shown to promote host adaptation [40], [41] and increase virulence [42], [43]. Conversely, mutation in amino acid 591 can reduce the selective pressure for mutations at amino acid 627, serving as an alternate human adaptive strategy [44]. This possible interaction is emphasised by the juxtaposition of residues 591 and 627, as observed in Figure 6. Of the remaining selected amino acids, some refer to mutations that can alter domain structure, three of which are human discriminating (661, 674, and 702). Interestingly, only one of the selected sites (292) differentiates canine viruses from equine viruses. The paired mean distance between groups (measured in terms of the number of differences observed in the full gene sequences) is smallest for the canine and equine viruses (Table S10). These host species turn out to be the ones with the most recent common ancestor [45], [46], lending additional support to the hypothesis that host phylogeny shapes evolution of viruses by affecting cross-species mutational barriers. However, influenza H1N1 viruses found in human hosts are more similar (on average) to avian viruses than to viruses found in other mammalian hosts. Bird viruses are also the least divergent comparison group from swine viruses, perhaps reflecting the avian origin of all influenza viruses, and that, for influenza, transmission between birds and some mammalian hosts (human and swine in this case) is more frequent than expected by their phylogenetic relationships, probably due to persistent exposure in domestic settings.

## Discussion

In recent years, genome-wide association studies (GWAS) have become an increasingly popular tool to identify genetic determinants of non-infectious human diseases [47]. However, statistically rigorous genotype-to-phenotype mapping for pathogens has been much less common. The methods used for human GWAS are particularly ill-suited to feature selection in RNA viruses, due to the short genome length, very high substitution rate and diversity, and the high degree of genetic linkage [48], [49]. Human GWAS tend to concentrate on common variants to explain the observed phenotypes [15], [49], [50] by looking at individual SNPs, thus having severe limitations in the presence of epistasis [15], [48], [50], [51]; our work demonstrates that non-parametric machine-learning based methods – such as RFA – are more appropriate in the context of RNA viruses, by identifying sets of substitutions associated with a particular phenotypic class, rather than solely evaluating the significance of individual polymorphisms [48], [51]. The incorporation of interactions among predictor variables in RFA makes it possible to identify possible epistatic effects, as highlighted in Figure 3, with substitutions being determinant for host discrimination when found together with other substitutions at other sites, but being fairly unimportant by themselves. While RFA and other related discriminative methods arise from a different theoretical paradigm from likelihood-based statistical models, their predictive performance can be readily assessed via bootstrapping and other resampling methods.

Our work demonstrates that machine-learning based feature selection methods are a powerful tool for *de novo* discovery of likely functional host discriminating markers, whilst providing a measure of the relative importance of those markers to host specificity. More generally, we highlight the potential of RFA for gaining important biological insights on cross-species transitions of RNA viruses.. First, we find that even relatively distantly related viruses within viral families – that might be geographically isolated and transmitted by different routes – share highly conserved genetic signatures of host specificity. Second, we see that the fitness landscapes of host adaptation are shaped by host phylogeny, with evolutionary barriers generally being lower between closely related host species, though not always (influenza A viruses transfer between birds and some mammalian hosts being a counter-example). Third, our analysis of influenza A often selects sites with empirically proven functional relevance [34], [36], [41], [44] to host specificity – in the case of HA, playing critical roles in cell receptor binding; for PB2, being exposed on the protein surface (Figure 6) and thus potentially interacting with host importin molecules to gain access to the nucleus [52] or with the nucleoprotein in the ribonucleoprotein complex [53], [54].

Overall, genotype to phenotype mapping using host reservoir as the discriminant phenotype can reveal evolutionary trajectories of RNA viruses in rapid expansion and under great evolutionary pressure (capturing the effects of diversification and expansion in a new host, as well as the contraction of diversity following host adaptation), while establishing the genetic signatures imposed by specific hosts which permit cross-species transmission events to be identified. Although discriminant analysis approaches are typically marred by biases related to sampling efforts and founder effects [55], RFA enables the circumvention of some of these biases through cross-validation, sampling with replacement and attribution of weights to unequally sampled groups (see Text S1 for more details). Even though some residual sources of bias are impossible to eliminate, these rigorous methods (which are computationally efficient and thus applicable to large numbers of sequences) are potentially useful for assessing the risk of viral emergence, and represent a powerful additional tool alongside phylogenetic analysis for analysing the phenotypic evolution of RNA viruses.

## Methods

### Feature selection algorithm

Feature selection methods try to find the subset of relevant features for building robust learning models that can accurately inform a classification algorithm [16]. We focussed on the random forest algorithm (RFA), since it offers excellent performance in classification tasks [17], and provides direct measures of variable importance and classification error. Each tree in a random forest is trained on a bootstrap sample of the data, and at each split a random subset of the variables is chosen from all the available variables (in this case, a subset of positions in the sequence for each split). Final classification of each sample results from aggregating the votes of all trees in the forest. The importance measure of each variable is obtained as the loss of accuracy of classification caused by the random permutation of attribute values for that variable. RFA identifies which variables give the most discriminating information regarding the independent categorical variable of interest (host reservoir in this case). We used the *varSelRF* package in *R* to run the random forest algorithm [56].

### Data preparation

The information within a given sequence alignment was numerically recoded into an allele frequency matrix, using the *adegenet* R package [57] (see Text S1 for more details). Starting from a multiple sequence alignment, all conserved sites are discarded, and a presence/absence matrix of all other alleles is assembled. Since we are dealing with RNA viruses, this matrix is actually equivalent to a presence/absence matrix of amino acid/nucleotide types in polymorphic sites (Table S1).

### Visualising the results

Outside of phylogenetic analysis, direct comparison of genetic sequences is challenging, due to the high dimensionality of the datasets, typically consisting of dozens of sequences containing thousands of nucleotides. However, the relationship between a set of viral sequences can be represented through dimensional reduction techniques such as principal component analysis (PCA) [58]. Here we use PCA simply as a tool to graphically represent the variance in our datasets and to highlight the relationships between the viral samples collected in different host species, similar to past studies [59]. Selecting the two dominant principal components (which in our study always explained more than 40% of the variance) allows for a straightforward interpretation of differences between any set of sequences through a two dimensional plot, with the scores for the two principal components serving as the coordinates. We can then assess how well feature selection clusters RNA viruses by phenotype class (here host reservoir) by applying PCA to both the original dataset and to the dataset consisting exclusively of sites selected by feature selection.

### Performance evaluation and solution stability

RFA prediction errors and variable importance are estimated from the samples which are left out of the training set at each split of the tree –the ‘out-of-bag’ samples. This makes RFA highly robust to over-fitting. Although RFA is unlikely to over-fit, we carried out cross-validation of the algorithm by performing multiple bootstrap runs of the feature selection procedure. Each bootstrap run is a new realisation of the complete feature selection procedure, thus removing selection bias concerns on the importance of the most significant variables.

More details on the methods employed throughout can be found in Text S1.

## Supporting Information

Figure S1 RFA error rates as a function of the number of variables in the forest (panels on the left) and solution stability (panels on the right) for 4 viral taxa. Points in the panels on the right reflect the proportion of trees in which the variable of the rank given on the x-axis from the original random forest are included among the top ranked X variables (X = 10 for blue points and X = 30 for red points) in the 100 bootstrap samples.
(TIF)

Figure S2 RFA error rates as a function of the number of variables in the forest (panels on the left) and solution stability (panels on the right) for 4 viral taxa. Points in the panels on the right reflect the proportion of trees in which the variable of the rank given on the x-axis from the original random forest are included among the top ranked X variables (X = 10 for blue and X = 30 for red points in the Rabies and SARS RFA runs; X = 25 for blue and X = 100 for red points in the influenza runs) in the 100 bootstrap samples.
(TIF)

Figure S3 Feature selection impact on host reservoir clustering, training set (TS) and out-of-bag samples (OOB) error rates for the viruses of 3 taxa (excluding Flaviviruses) discriminated in Table S1. We display the relationship between viral sequences according to the scores of the first two principal components of the PCA analysis of both the original sequences and the sequences containing only those amino acids which were selected by feature selection. Colour coding of host reservoir is as follows: gold – primates/humans; purple – birds; green – other mammals/artiodactyls; red – bats/carnivores; blue – rodents/lagomorphs.
(TIF)

Figure S4 Bat species phylogeny according to the 12S ribosomal RNA gene (Genbank reference for sequences - AF263219, AF326092, AY495480, AY495484, and AY495482). The maximum likelihood tree is shown, displaying the percentage of trees in which the associated taxa clustered together next to the branches. The tree is drawn to scale, with branch lengths measured in number of substitutions per site. All positions containing gaps and missing data were eliminated. There were a total of 1014 positions in the final dataset.
(TIF)

Figure S5 Variable importance scores obtained from the RFA when using only viruses from the Human and Swine groups and all the samples (Human, Swine and pandemic Human groups). The different colors discriminate the 4 groups defined by k-means clustering.
(TIF)

Table S1 Representation of the genetic data matrixes used to run the RFA. From a starting multiple sequence alignment (A) we discard all non-polymorphic sites (marked by asterisks), and build a presence/absence matrix of all other alleles as in Table S1B.
(DOCX)

Table S2 Polymerase gene sequences used to analyse RNA viruses of several taxa. Sources are provided for the natural host reservoir classification.
(DOCX)

Table S3 SARS coronavirus sequences used.
(DOCX)

Table S4 SARS-like virus nucleotide variants present in the feature selected alleles and corresponding amino acid residues. Putative residue positions in the 3D conformation of the spike protein were suggested by [22]. Synonymous substitutions are shown in italic.
(DOCX)

Table S5 Rabies virus nucleoprotein sequences analysed.
(DOCX)

Table S6 Random forest host reservoir prediction probabilities for rabies viruses, excluding the putative species transition samples.
(DOCX)

Table S7 Positions selected as host-specific in the influenza A HA analysis. The positions are ordered according to their predicted RFA importance for classification.
(DOCX)

Table S8 Sequences used for the analysis of the influenza A PB2 segment by subtype and host reservoir.
(DOCX)

Table S9 Influenza virus host reservoir relevant amino acids and their respective level of conservation across all viral subtypes (δh, δa, δs, δc, and δe).
(DOCX)

Table S10 Mean pairwise distance (measured in terms of amino acid differences) between and within host reservoir groups for the influenza viruses used in the PB2 analysis.
(DOCX)

Table S11 Summary of the classification type random forest algorithms performed and overall prediction error.
(DOCX)

Text S1 Includes a more detailed description of the methods used throughout, as well as Figures S1, S2, S3, S4, S5 and Tables S1, S2, S3, S4, S5, S6, S7, S8, S9, S10, S11.
(DOCX)
