# Supplementary figures and images for: Feature Selection Methods for Identifying Genetic Determinants of Host Species in RNA Viruses
Source: PLoS Comput Biol. 2013 Oct 10;9(10):e1003254. doi: 10.1371/journal.pcbi.1003254 (PMC3794897; doi:10.1371/journal.pcbi.1003254)

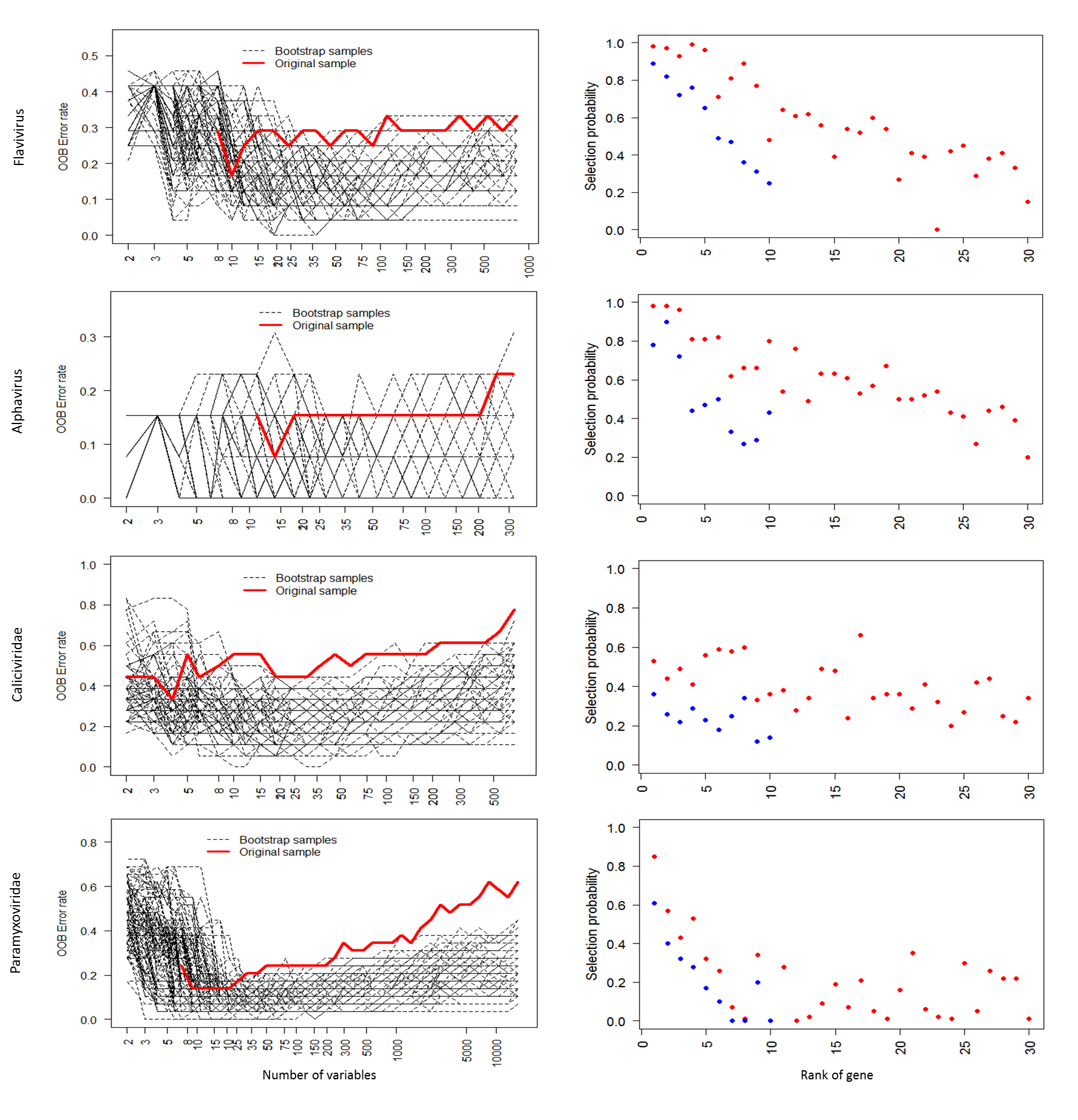

Supplement: Figure S1 — RFA error rates as a function of the number of variables in the forest (panels on the left) and solution stability (panels on the right) for 4 viral taxa. Points in the panels on the right reflect the proportion of trees in which the variable of the rank given on the x-axis from the original random forest are included among the top ranked X variables (X = 10 for blue points and X = 30 for red points) in the 100 bootstrap samples. (TIF) [file pcbi.1003254.s001.tif]

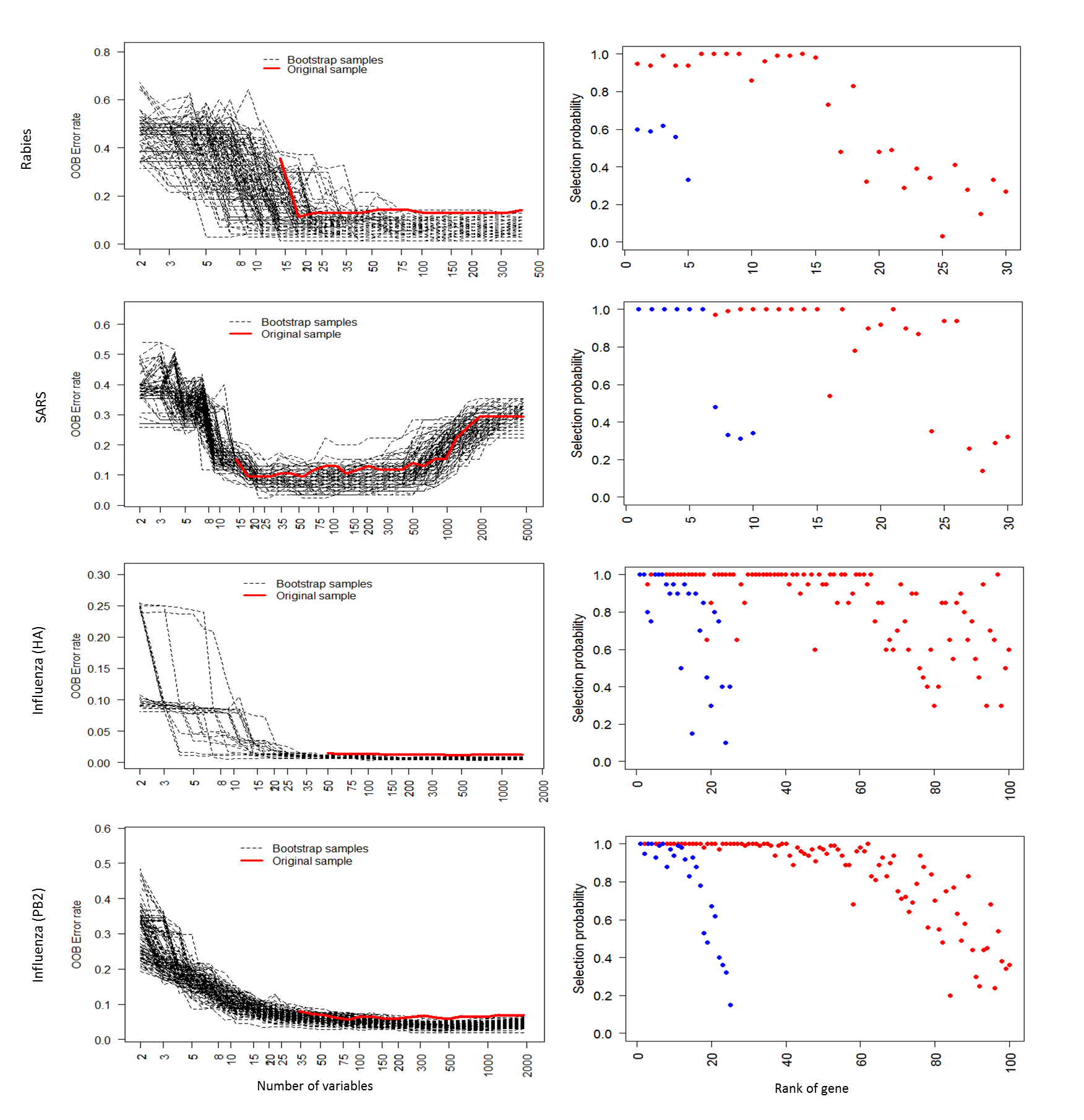

Supplement: Figure S2 — RFA error rates as a function of the number of variables in the forest (panels on the left) and solution stability (panels on the right) for 4 viral taxa. Points in the panels on the right reflect the proportion of trees in which the variable of the rank given on the x-axis from the original random forest are included among the top ranked X variables (X = 10 for blue and X = 30 for red points in the Rabies and SARS RFA runs; X = 25 for blue and X = 100 for red points in the influenza runs) in the 100 bootstrap samples. (TIF) [file pcbi.1003254.s002.tif]

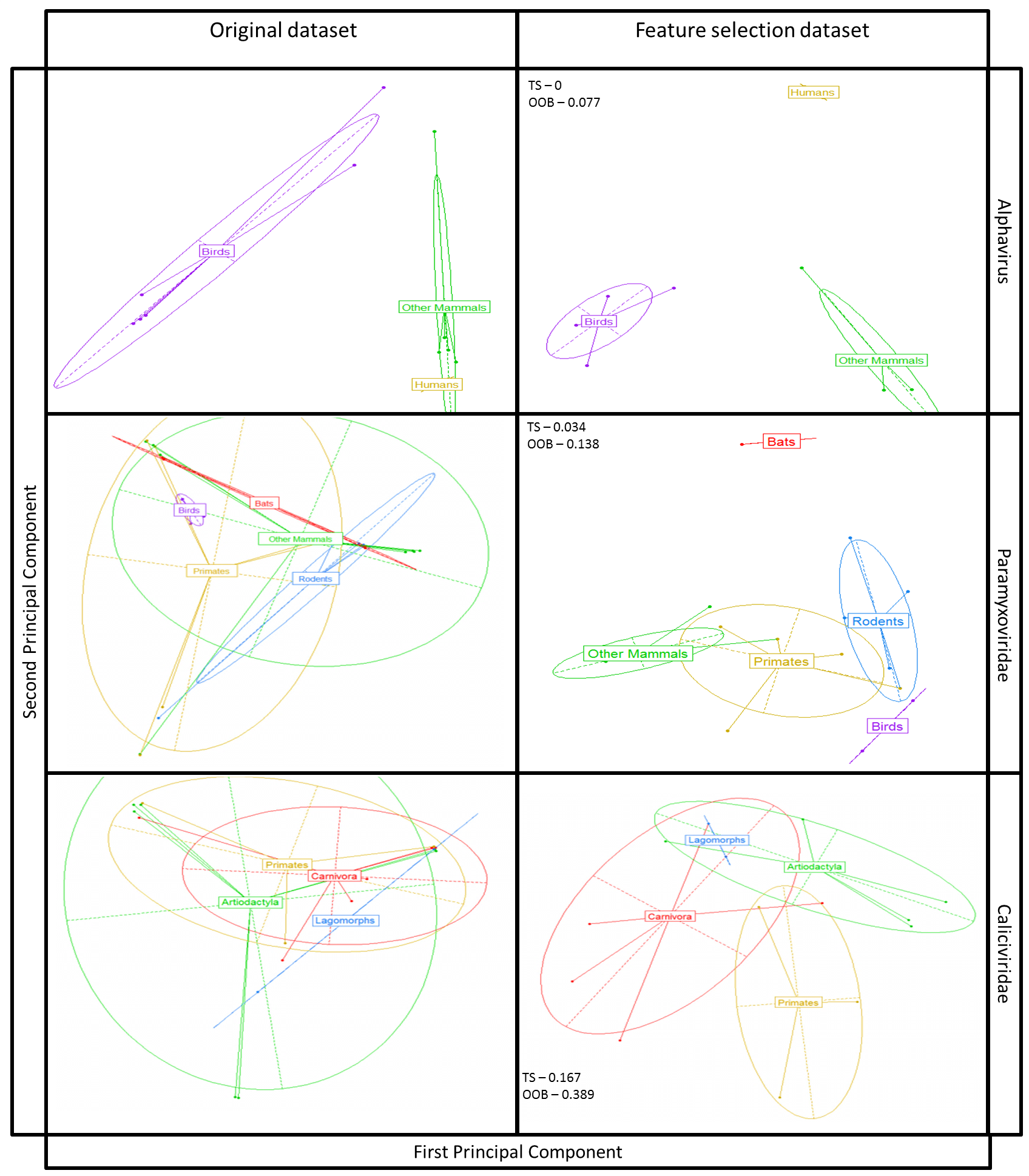

Supplement: Figure S3 — Feature selection impact on host reservoir clustering, training set (TS) and out-of-bag samples (OOB) error rates for the viruses of 3 taxa (excluding Flaviviruses) discriminated in Table S1. We display the relationship between viral sequences according to the scores of the first two principal components of the PCA analysis of both the original sequences and the sequences containing only those amino acids which were selected by feature selection. Colour coding of host reservoir is as follows: gold – primates/humans; purple – birds; green – other mammals/artiodactyls; red – bats/carnivores; blue – rodents/lagomorphs. (TIF) [file pcbi.1003254.s003.tif]

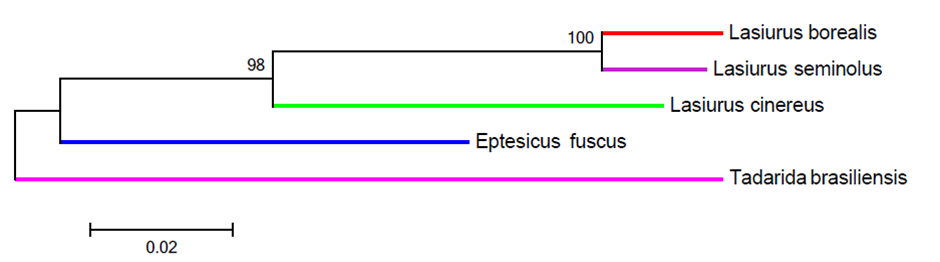

Supplement: Figure S4 — Bat species phylogeny according to the 12S ribosomal RNA gene (Genbank reference for sequences - AF263219, AF326092, AY495480, AY495484, and AY495482). The maximum likelihood tree is shown, displaying the percentage of trees in which the associated taxa clustered together next to the branches. The tree is drawn to scale, with branch lengths measured in number of substitutions per site. All positions containing gaps and missing data were eliminated. There were a total of 1014 positions in the final dataset. (TIF) [file pcbi.1003254.s004.tif]

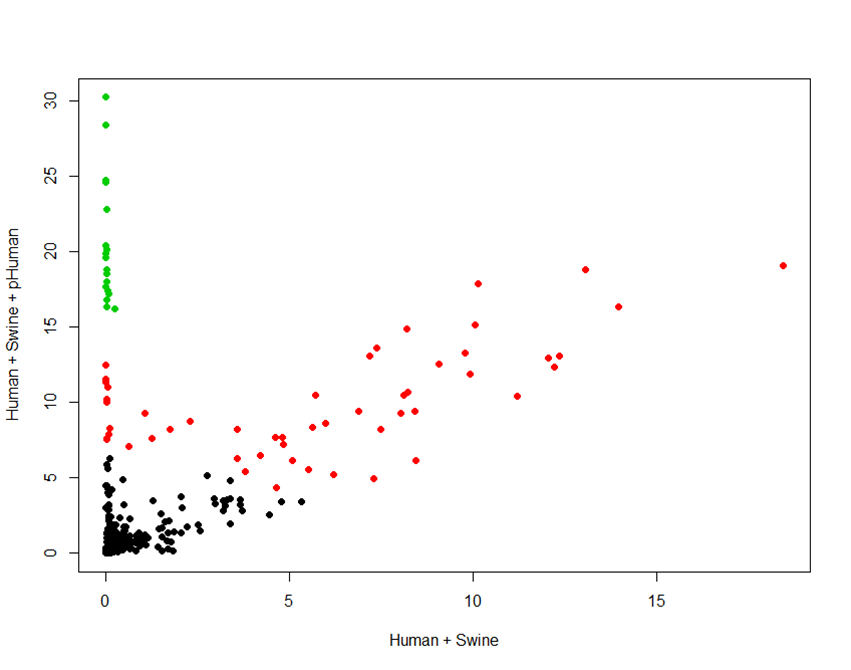

Supplement: Figure S5 — Variable importance scores obtained from the RFA when using only viruses from the Human and Swine groups and all the samples (Human, Swine and pandemic Human groups). The different colors discriminate the 4 groups defined by k-means clustering. (TIF) [file pcbi.1003254.s005.tif]
