# Supplementary material for: Feature Selection Methods for Identifying Genetic Determinants of Host Species in RNA Viruses
Source: PLoS Comput Biol. 2013 Oct 10;9(10):e1003254. doi: 10.1371/journal.pcbi.1003254 (PMC3794897; doi:10.1371/journal.pcbi.1003254)

Table S1. Representation of the genetic data matrixes used to run the RFA. From a starting multiple sequence alignment (A) we discard all non-polymorphic sites (marked by asterisks), and build a presence/absence matrix of all other alleles as in Table S1B.


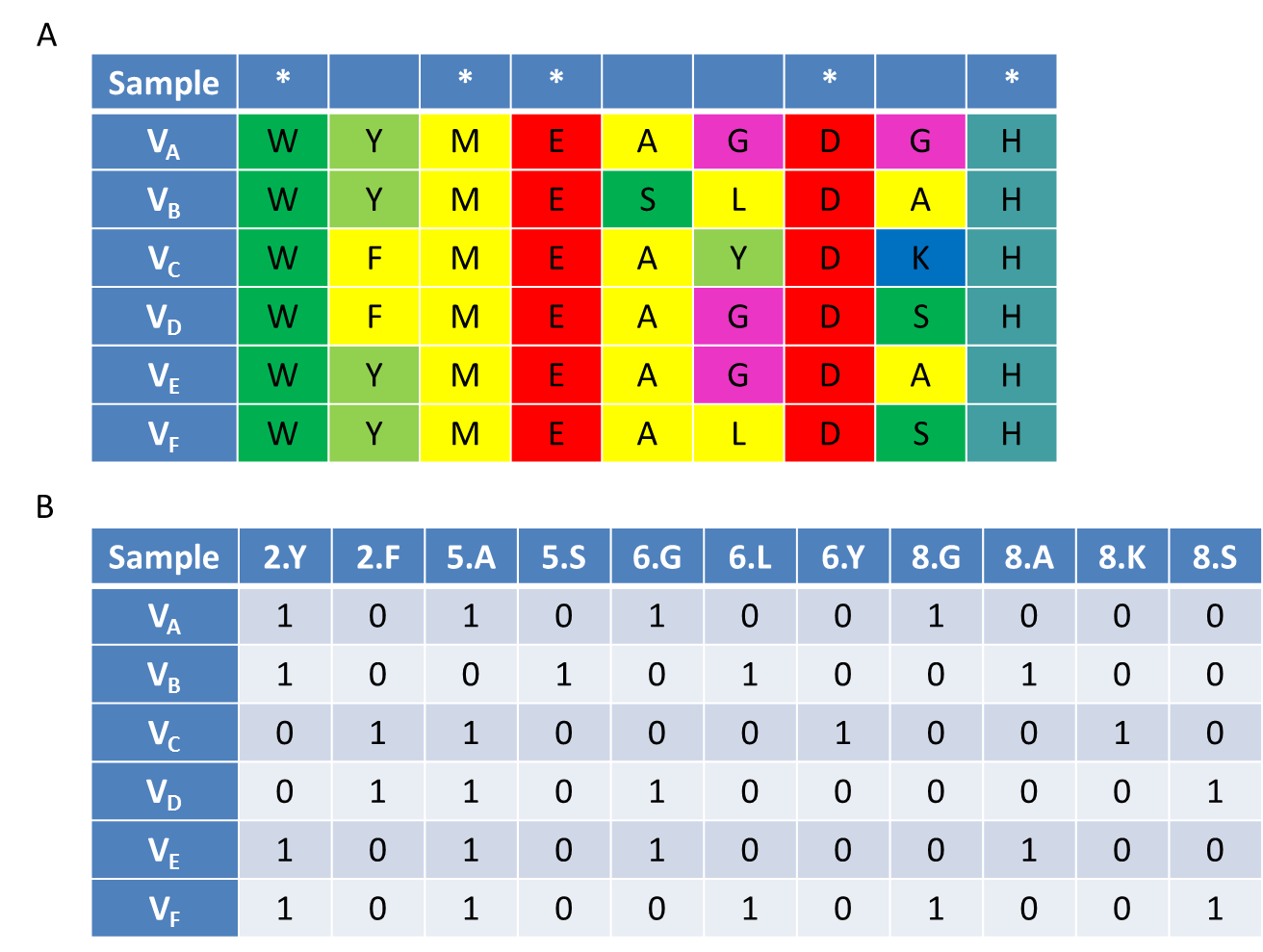

Supplement: Table S1 — Representation of the genetic data matrixes used to run the RFA. From a starting multiple sequence alignment (A) we discard all non-polymorphic sites (marked by asterisks), and build a presence/absence matrix of all other alleles as in Table S1B. (DOCX) [file pcbi.1003254.s006.docx]
