# Supplementary material for: Feature Selection Methods for Identifying Genetic Determinants of Host Species in RNA Viruses
Source: PLoS Comput Biol. 2013 Oct 10;9(10):e1003254. doi: 10.1371/journal.pcbi.1003254 (PMC3794897; doi:10.1371/journal.pcbi.1003254)
Supplement: Table S2 — Polymerase gene sequences used to analyse RNA viruses of several taxa. Sources are provided for the natural host reservoir classification. (DOCX) [file pcbi.1003254.s007.docx]

Table S2. Polymerase gene sequences used to analyse RNA viruses of several taxa. Sources are provided for the natural host reservoir classification.

| Species | Genbank reference | Natural reservoir | Source |
| --- | --- | --- | --- |
| Flavivirus | | | |
| Alkhurma virus | NP_775478 | Rodents | [[1](#_ENREF_1)] |
| Apoi virus | AAC58749 | Rodents | [[2](#_ENREF_2)] |
| Aroa virus | YP_001040004 | Rodents | [[3](#_ENREF_3)] |
| Bagaza virus | AAC58751 | Birds | [[4](#_ENREF_4)] |
| Dengue | AAT07549 | Primates | [[5](#_ENREF_5)] |
| Entebbe bat virus | AAC58761 | Bats | [[6](#_ENREF_6)] |
| Ilheus virus | ABQ88008 | Birds | [[7](#_ENREF_7)] |
| Japanese encephalitis virus | NP_775674 | Birds | [[8](#_ENREF_8)] |
| Kokobera virus | AAC58771 | Other Mammals | [[9](#_ENREF_9)] |
| Langat virus | NP_740302 | Other Mammals | [[10](#_ENREF_10)] |
| Louping ill virus | NP_740729 | Other Mammals | [[11](#_ENREF_11)] |
| Modoc virus | AAC58775 | Rodents | [[12](#_ENREF_12)] |
| Murray Valley encephalitis virus | AAC58777 | Birds | [[13](#_ENREF_13)] |
| Omsk virus | AAC58781 | Rodents | [[14](#_ENREF_14)] |
| Powassan virus | NP_775524 | Rodents | [[15](#_ENREF_15)] |
| Rio bravo virus | AAC58784 | Bats | [[16](#_ENREF_16)] |
| Sepik virus | AAC58792 | Primates | [[17](#_ENREF_17)] |
| St Louis encephalitis | AAB96583 | Birds | [[18](#_ENREF_18)] |
| Tick-borne Encephalitis virus | NP_775511 | Rodents | [[19](#_ENREF_19)] |
| Usutu virus | AAC58800 | Birds | [[20](#_ENREF_20)] |
| West Nile Virus | ADG27877 | Birds | [[18](#_ENREF_18),[21](#_ENREF_21)] |
| Yellow fever virus | AAC58804 | Primates | [[22](#_ENREF_22)] |
| Yokose virus | AAC58802 | Bats | [[23](#_ENREF_23)] |
| Zika virus | AAC58803 | Primates | [[24](#_ENREF_24)] |
| Alphavirus | | | |
| Aura virus | NP_819013 | Birds | [[25](#_ENREF_25)] |
| Barmah Forest virus | NP_597797 | Other mammals | [[26](#_ENREF_26)] |
| Eastern equine encephalitis | NP_740652 | Birds | [[27](#_ENREF_27),[28](#_ENREF_28)] |
| Chikungunya virus | ADZ47898 | Humans | [[29](#_ENREF_29)] |
| Getah virus | ABR23662 | Other mammals | [[30](#_ENREF_30)] |
| Mayaro virus | NP_740690 | Other mammals | [[31](#_ENREF_31)] |
| O'nyong-nyong virus | NP_740706 | Humans | [[32](#_ENREF_32)] |
| Ross river virus | NP_740681 | Other mammals | [[26](#_ENREF_26)] |
| Semliki forest virus | NP_740668 | Other mammals | [[33](#_ENREF_33)] |
| Sindbis virus | NP_740669 | Birds | [[27](#_ENREF_27)] |
| Highlands J virus | YP_002802304 | Birds | [[34](#_ENREF_34)] |
| Fort Morgan virus | YP_003324594 | Birds | [[35](#_ENREF_35)] |
| Western equine encephalomyelitis virus | NP_818936 | Birds | [[27](#_ENREF_27),[36](#_ENREF_36)] |
| Paramixoviridae |  |  |  |
| Avian paramyxovirus 2 | ADK_25248 | Birds | [[37](#_ENREF_37)] |
| Avian paramyxovirus 3 | ACI47553 | Birds | [[37](#_ENREF_37)] |
| Beilong virus | YP_512254 | Rodents | [[38](#_ENREF_38)] |
| Bovine parainfluenza virus | ADQ43756 | Other Mammals | [[39](#_ENREF_39)] |
| Bovine respiratory syncytial virus | NP_048058 | Other Mammals | [[40](#_ENREF_40)] |
| Canine distemper virus | AAR32274 | Other Mammals | [[41](#_ENREF_41)] |
| Dolphin morbillivirus | NP_945030 | Other Mammals | [[42](#_ENREF_42)] |
| Goose paramyxovirus | NP_872278 | Birds | [[43](#_ENREF_43)] |
| Hendra virus | NP_047113 | Bats | [[44](#_ENREF_44)] |
| Human metapneumovirus | YP_012613 | Primates | [[45](#_ENREF_45)] |
| Human parainfluenza virus 1 | NP_604442 | Primates | [[46](#_ENREF_46)] |
| Human parainfluenza virus 2 | NP_598406 | Primates | [[46](#_ENREF_46)] |
| Human parainfluenza virus 3 | AAB48690 | Primates | [[46](#_ENREF_46)] |
| Human respiratory syncytial virus | AAB82445 | Primates | [[46](#_ENREF_46)] |
| J-virus | YP_338085 | Primates | [[47](#_ENREF_47)] |
| Menangle virus | YP_415514 | Bats | [[48](#_ENREF_48)] |
| Mossman virus | NP_958055 | Rodents | [[49](#_ENREF_49)] |
| Mumps virus | AAL83746 | Primates | [[50](#_ENREF_50)] |
| Murine pneumonia virus | AAW02843 | Rodents | [[51](#_ENREF_51)] |
| Newcastle disease virus | AAK55552 | Birds | [[52](#_ENREF_52)] |
| Nipah virus | NP_112028 | Bats | [[44](#_ENREF_44)] |
| Peste-des-petits-ruminants virus | CAD88265 | Other Mammals | [[53](#_ENREF_53)] |
| Phocine distemper virus | CAA70843 | Other Mammals | [[54](#_ENREF_54)] |
| Porcine rubulavirus | CAA66807 | Other Mammals | [[55](#_ENREF_55)] |
| Rinderpest virus | YP087126 | Other Mammals | [[56](#_ENREF_56)] |
| Sendai virus | NP_056879 | Rodents | [[57](#_ENREF_57)] |
| Simian Parainfluenza virus 5 | YP_138518 | Other Mammals | [[58](#_ENREF_58)] |
| Simian virus 41 | YP_138510 | Primates | [[59](#_ENREF_59)] |
| Tioman virus | NP665871 | Bats | [[60](#_ENREF_60)] |
| Caliciviridae | | | |
| Bovine calicivirus | CAA09480 | Artiodactyla | [[61](#_ENREF_61)] |
| Bovine enteric calicivirus | AAP83352 | Artiodactyla | [[62](#_ENREF_62)] |
| Canine calicivirus | AAC16445 | Carnivores | [[63](#_ENREF_63)] |
| European brown hare syndrome virus | NP_786902 | Lagomorphs | [[64](#_ENREF_64)] |
| Feline calicivirus | NP_783310 | Carnivores | [[65](#_ENREF_65)] |
| Mink enteric sapovirus | AAN64326 | Carnivores | [[66](#_ENREF_66)] |
| Nebraska-like virus | NP_663315 | Artiodactyla | [[61](#_ENREF_61)] |
| Newbury agent 1 | YP_529897 | Artiodactyla | [[67](#_ENREF_67)] |
| Norovirus genogroup II (swine calicivirus) | BAB83513 | Artiodactyla | [[68](#_ENREF_68)] |
| Norovirus genogroup IV (lion) | ABR15782 | Carnivores | [[69](#_ENREF_69)] |
| Rabbit vesivirus | YP_873922 | Lagomorphs | [[70](#_ENREF_70)] |
| San miguel sea lion virus | ABI34890 | Artiodactyla | [[71](#_ENREF_71)] |
| Sapovirus genogroup I (Sapporo) | AAC40578 | Primates | [[72](#_ENREF_72)] |
| Steller sea lion vesivirus | YP_002004564 | Carnivores | [[73](#_ENREF_73)] |
| Vesicular exanthema of swine virus | NP_786896 | Artiodactyla | [[74](#_ENREF_74)] |
| Walrus calicivirus | NP_786919 | Carnivores | [[75](#_ENREF_75)] |
| Norovirus genogroup II (Lordsdale) | ABA12125 | Primates | [[76](#_ENREF_76)] |
| Primate calicivirus virus/VESV-like/Pan-1 | AAC61758 | Primates | [[77](#_ENREF_77)] |

**References**

1. Charrel RN, Fagbo S, Moureau G, Alqahtani MH, Temmam S, et al. (2007) Alkhurma hemorrhagic fever virus in Ornithodoros savignyi ticks. Emerg Infect Dis 13: 153-155.

2. Varelas-Wesley I, Calisher CH (1982) Antigenic relationships of flaviviruses with undetermined arthropod-borne status. Am J Trop Med Hyg 31: 1273-1284.

3. Weissenbock H, Hubalek Z, Bakonyi T, Nowotny N (2010) Zoonotic mosquito-borne flaviviruses: Worldwide presence of agents with proven pathogenicity and potential candidates of future emerging diseases. Veterinary Microbiology 140: 271-280.

4. Bondre VP, Sapkal GN, Yergolkar PN, Fulmali PV, Sankararaman V, et al. (2009) Genetic characterization of Bagaza virus (BAGV) isolated in India and evidence of anti-BAGV antibodies in sera collected from encephalitis patients. Journal of General Virology 90: 2644-2649.

5. Gubler DJ (2001) Epidemic dengue/dengue haemorrhagic fever as a public health problem in the 21st century. New Challenges to Health: The Threat of Virus Infection 60: 247-267.

6. Kuno G, Chang GJ (2006) Characterization of Sepik and Entebbe bat viruses closely related to yellow fever virus. Am J Trop Med Hyg 75: 1165-1170.

7. Pereira LE, Suzuki A, Coimbra TL, de Souza RP, Chamelet EL (2001) [Ilheus arbovirus in wild birds (Sporophila caerulescens and Molothrus bonariensis)]. Rev Saude Publica 35: 119-123.

8. Vaughn DW, Hoke CH (1992) The Epidemiology of Japanese Encephalitis - Prospects for Prevention. Epidemiologic Reviews 14: 197-221.

9. Doherty RL, Standfas.Ha, Domrow R, Wetters EJ, Whitehea.Rh, et al. (1971) Epidemiology of Arthropod-Borne Virus Infections at Mitchell River Mission, Cape York Peninsula, North-Queensland .4. Arbovirus Infections of Mosquitoes and Mammals, 1967-1969. Transactions of the Royal Society of Tropical Medicine and Hygiene 65: 504-&.

10. Smith CE (1956) A virus resembling Russian spring-summer encephalitis virus from an ixodid tick in Malaya. Nature 178: 581-582.

11. Gould EA, McGuire K, Holmes EC, Gao GF, Reid HW (1998) Tracing the origins of louping ill virus by molecular phylogenetic analysis. Journal of General Virology 79: 981-988.

12. Davis JW, Hardy JL, Reeves WC (1974) Modoc Viral-Infections in Deer Mouse Peromyscus-Maniculatus. Infection and Immunity 10: 1362-1369.

13. Marshall ID, Brown BK, Keith K, Gard GP, Thibos E (1982) Variation in arbovirus infection rates in species of birds sampled in a serological survey during an encephalitis epidemic in the Murray Valley of South-eastern Australia, February 1974. Aust J Exp Biol Med Sci 60 (Pt 5): 471-478.

14. Ruzek D, Yakimenko VV, Karan LS, Tkachev SE (2010) Omsk haemorrhagic fever. Lancet 376: 2104-2113.

15. McLean DM, Walker SJ, Macpherson LW, Scholten TH, Ronald K, et al. (1961) Powassan virus: investigations of possible natural cycles of infection. J Infect Dis 109: 19-23.

16. Constantine DG, Woodall DF (1964) Latent Infection of Rio Bravo Virus in Salivary Glands of Bats. Public Health Rep 79: 1033-1039.

17. Mackenzie JS, Lindsay MD, Coelen RJ, Broom AK, Hall RA, et al. (1994) Arboviruses Causing Human-Disease in the Australasian Zoogeographic Region. Archives of Virology 136: 447-467.

18. Reisen WK, Fang Y, Martinez VM (2005) Avian host and mosquito (Diptera : Culicidae) vector competence determine the efficiency of west nile and St. Louis encephalitis virus transmission. Journal of Medical Entomology 42: 367-375.

19. Randolph SE, Miklisova D, Lysy J, Rogers DJ, Labuda M (1999) Incidence from coincidence: patterns of tick infestations on rodents facilitate transmission of tick-borne encephalitis virus. Parasitology 118: 177-186.

20. Weissenbock H, Kolodziejek J, Url A, Lussy H, Rebel-Bauder B, et al. (2002) Emergence of Usutu virus, an African mosquito-borne flavivirus of the Japanese encephalitis virus group, central Europe. Emerg Infect Dis 8: 652-656.

21. Malkinson M, Banet C (2002) The role of birds in the ecology of West Nile virus in Europe and Africa. Japanese Encephalitis and West Nile Viruses 267: 309-322.

22. Gould EA, Zanotto PMD, Holmes EC (1997) The genetic evolution of flaviviruses. Factors in the Emergence of Arbovirus Diseases: 51-63.

23. Kurane I, Tajima S, Takasaki T, Matsuno S, Nakayama M (2005) Genetic characterization of Yokose virus, a flavivirus isolated from the bat in Japan. Virology 332: 38-44.

24. Duffy MR, Chen TH, Hancock WT, Powers AM, Kool JL, et al. (2009) Zika virus outbreak on Yap Island, Federated States of Micronesia. N Engl J Med 360: 2536-2543.

25. Rumenapf T, Strauss EG, Strauss JH (1995) Aura Virus Is a New-World Representative of Sindbis-Like Viruses. Virology 208: 621-633.

26. Kay BH, Boyd AM, Ryan PA, Hall RA (2007) Mosquito feeding patterns and natural infection of vertebrates with Ross River and Barmah Forest viruses in Brisbane, Australia. Am J Trop Med Hyg 76: 417-423.

27. Yuill TM (1986) The Ecology of Tropical Arthropod-Borne Viruses. Annual Review of Ecology and Systematics 17: 189-219.

28. Estep LK, McClure CJ, Burkett-Cadena ND, Hassan HK, Hicks TL, et al. (2011) A multi-year study of mosquito feeding patterns on avian hosts in a southeastern focus of eastern equine encephalitis virus. Am J Trop Med Hyg 84: 718-726.

29. Powers AM, Logue CH (2007) Changing patterns of chikungunya virus: re-emergence of a zoonotic arbovirus. J Gen Virol 88: 2363-2377.

30. Kumanomido T, Fukunaga Y, Kamada M, Imagawa H, Ando Y, et al. (1986) Getah virus isolations from mosquitoes collected at two horse habitations in the western areas of Japan. Nihon Juigaku Zasshi 48: 1191-1197.

31. Powers AM, Aguilar PV, Chandler LJ, Brault AC, Meakins TA, et al. (2006) Genetic relationships among Mayaro and Una viruses suggest distinct patterns of transmission. Am J Trop Med Hyg 75: 461-469.

32. Powers AM, Brault AC, Tesh RB, Weaver SC (2000) Re-emergence of Chikungunya and O'nyong-nyong viruses: evidence for distinct geographical lineages and distant evolutionary relationships. J Gen Virol 81: 471-479.

33. Fazakerley JK (2002) Pathogenesis of Semliki Forest virus encephalitis. J Neurovirol 8 Suppl 2: 66-74.

34. McLean RG, Frier G, Parham GL, Francy DB, Monath TP, et al. (1985) Investigations of the vertebrate hosts of eastern equine encephalitis during an epizootic in Michigan, 1980. Am J Trop Med Hyg 34: 1190-1202.

35. Calisher CH, Monath TP, Muth DJ, Lazuick JS, Trent DW, et al. (1980) Characterization of Fort Morgan virus, an alphavirus of the western equine encephalitis virus complex in an unusual ecosystem. Am J Trop Med Hyg 29: 1428-1440.

36. Reisen WK, Chiles RE, Martinez VM, Fang Y, Green EN (2004) Encephalitis virus persistence in California birds: experimental infections in mourning doves (Zenaidura macroura). Journal of Medical Entomology 41: 462-466.

37. Andral B, Toquin D (1984) Isolation of avian paramyxovirus 2 and 3 from turkeys in Brittany. Vet Rec 114: 570-571.

38. Li Z, Yu M, Zhang H, Magoffin DE, Jack PJ, et al. (2006) Beilong virus, a novel paramyxovirus with the largest genome of non-segmented negative-stranded RNA viruses. Virology 346: 219-228.

39. Schmidt AC, McAuliffe JM, Huang A, Surman SR, Bailly JE, et al. (2000) Bovine parainfluenza virus type 3 (BPIV3) fusion and hemagglutinin-neuraminidase glycoproteins make an important contribution to the restricted replication of BPIV3 in primates. J Virol 74: 8922-8929.

40. Valarcher JF, Schelcher F, Bourhy H (2000) Evolution of bovine respiratory syncytial virus. J Virol 74: 10714-10728.

41. Almberg ES, Cross PC, Smith DW (2010) Persistence of canine distemper virus in the Greater Yellowstone ecosystem's carnivore community. Ecol Appl 20: 2058-2074.

42. Van Bressem M, Waerebeek KV, Jepson PD, Raga JA, Duignan PJ, et al. (2001) An insight into the epidemiology of dolphin morbillivirus worldwide. Veterinary Microbiology 81: 287-304.

43. Zou J, Shan S, Yao N, Gong Z (2005) Complete genome sequence and biological characterizations of a novel goose paramyxovirus-SF02 isolated in China. Virus Genes 30: 13-21.

44. Eaton BT, Broder CC, Middleton D, Wang LF (2006) Hendra and Nipah viruses: different and dangerous. Nat Rev Microbiol 4: 23-35.

45. Kahn JS (2006) Epidemiology of human metapneumovirus. Clin Microbiol Rev 19: 546-557.

46. Hall CB (2001) Respiratory syncytial virus and parainfluenza virus. N Engl J Med 344: 1917-1928.

47. Jun MH, Karabatsos N, Johnson RH (1977) A new mouse paramyxovirus (J virus). Aust J Exp Biol Med Sci 55: 645-647.

48. Bowden TR, Westenberg M, Wang LF, Eaton BT, Boyle DB (2001) Molecular characterization of Menangle virus, a novel paramyxovirus which infects pigs, fruit bats, and humans. Virology 283: 358-373.

49. Miller PJ, Boyle DB, Eaton BT, Wang LF (2003) Full-length genome sequence of Mossman virus, a novel paramyxovirus isolated from rodents in Australia. Virology 317: 330-344.

50. Hviid A, Rubin S, Muhlemann K (2008) Mumps. Lancet 371: 932-944.

51. Krempl CD, Wnekowicz A, Lamirande EW, Nayebagha G, Collins PL, et al. (2007) Identification of a novel virulence factor in recombinant pneumonia virus of mice. Journal of Virology 81: 9490-9501.

52. Cattoli G, Susta L, Terregino C, Brown C (2011) Newcastle disease: a review of field recognition and current methods of laboratory detection. J Vet Diagn Invest 23: 637-656.

53. Kwiatek O, Ali YH, Saeed IK, Khalafalla AI, Mohamed OI, et al. (2011) Asian lineage of peste des petits ruminants virus, Africa. Emerg Infect Dis 17: 1223-1231.

54. Nielsen L, Arctander P, Jensen TH, Dietz HH, Hammer AS, et al. (2009) Genetic diversity and phylogenetic analysis of the attachment glycoprotein of phocine distemper viruses of the 2002 and 1988 epizootics. Virus Res 144: 323-328.

55. Wang LF, Hansson E, Yu M, Chua KB, Mathe N, et al. (2007) Full-length genome sequence and genetic relationship of two paramyxoviruses isolated from bat and pigs in the Americas. Arch Virol 152: 1259-1271.

56. Morens DM, Holmes EC, Davis AS, Taubenberger JK (2011) Global rinderpest eradication: lessons learned and why humans should celebrate too. J Infect Dis 204: 502-505.

57. Iida T, Tajima M, Murata Y (1973) Transmission of maternal antibodies to Sendai virus in mice and its significance in enzootic infection. J Gen Virol 18: 247-254.

58. Chatziandreou N, Stock N, Young D, Andrejeva J, Hagmaier K, et al. (2004) Relationships and host range of human, canine, simian and porcine isolates of simian virus 5 (parainfluenza virus 5). J Gen Virol 85: 3007-3016.

59. Nishio M, Tsurudome M, Bando H, Ito Y (1990) Immunological relationships of simian virus 41 (SV41) to other paramyxoviruses and serological evidence of SV41 infection in human populations. J Gen Virol 71 ( Pt 9): 2093-2097.

60. Chua KB, Wang LF, Lam SK, Crameri G, Yu M, et al. (2001) Tioman virus, a novel paramyxovirus isolated from fruit bats in Malaysia. Virology 283: 215-229.

61. Smiley JR, Chang KO, Hayes J, Vinje J, Saif LJ (2002) Characterization of an enteropathogenic bovine calicivirus representing a potentially new calicivirus genus. J Virol 76: 10089-10098.

62. Liu BL, Lambden PR, Gunther H, Otto P, Elschner M, et al. (1999) Molecular characterization of a bovine enteric calicivirus: relationship to the Norwalk-like viruses. J Virol 73: 819-825.

63. Matsuura Y, Tohya Y, Nakamura K, Shimojima M, Roerink F, et al. (2002) Complete nucleotide sequence, genome organization and phylogenic analysis of the canine calicivirus. Virus Genes 25: 67-73.

64. Drews B, Szentiks CA, Roellig K, Fickel J, Schroeder K, et al. (2011) Epidemiology, control and management of an EBHS outbreak in captive hares. Veterinary Microbiology.

65. Coyne KP, Edwards D, Radford AD, Cripps P, Jones D, et al. (2007) Longitudinal molecular epidemiological analysis of feline calicivirus infection in an animal shelter: a model for investigating calicivirus transmission within high-density, high-turnover populations. J Clin Microbiol 45: 3239-3244.

66. Guo M, Evermann JF, Saif LJ (2001) Detection and molecular characterization of cultivable caliciviruses from clinically normal mink and enteric caliciviruses associated with diarrhea in mink. Arch Virol 146: 479-493.

67. Bridger JC, Hall GA, Brown JF (1984) Characterization of a calici-like virus (Newbury agent) found in association with astrovirus in bovine diarrhea. Infection and Immunity 43: 133-138.

68. Sugieda M, Nagaoka H, Kakishima Y, Ohshita T, Nakamura S, et al. (1998) Detection of Norwalk-like virus genes in the caecum contents of pigs. Arch Virol 143: 1215-1221.

69. Martella V, Campolo M, Lorusso E, Cavicchio P, Camero M, et al. (2007) Norovirus in captive lion cub (Panthera leo). Emerg Infect Dis 13: 1071-1073.

70. Martin-Alonso JM, Skilling DE, Gonzalez-Molleda L, del Barrio G, Machin A, et al. (2005) Isolation and characterization of a new Vesivirus from rabbits. Virology 337: 373-383.

71. Wellehan JFX, Yu FH, Venn-Watson SK, Jensen ED, Smith CR, et al. (2010) Characterization of San Miguel Sea Lion Virus populations using pyrosequencing-based methods. Infection Genetics and Evolution 10: 254-260.

72. Kapikian AZ, Wyatt RG, Dolin R, Thornhill TS, Kalica AR, et al. (1972) Visualization by immune electron microscopy of a 27-nm particle associated with acute infectious nonbacterial gastroenteritis. J Virol 10: 1075-1081.

73. McClenahan SD, Burek KA, Beckmen KB, Knowles NJ, Neill JD, et al. (2008) Genomic characterization of novel marine vesiviruses from Steller sea lions (Eumetopias jubatus) from Alaska. Virus Res 138: 26-35.

74. Seal BS, House JA, Whetstone CA, Neill JD (1995) Analysis of the Serologic Relationship among San-Miguel Sea Lion Virus and Vesicular Exanthema of Swine Virus Isolates - Application of the Western-Blot Assay for Detection of Antibodies in Swine Sera to These Virus Types. Journal of Veterinary Diagnostic Investigation 7: 190-195.

75. Ganova-Raeva L, Smith AW, Fields H, Khudyakov Y (2004) New Calicivirus isolated from walrus. Virus Res 102: 207-213.

76. Dingle KE, Lambden PR, Caul EO, Clarke IN (1995) Human enteric Caliciviridae: the complete genome sequence and expression of virus-like particles from a genetic group II small round structured virus. J Gen Virol 76 ( Pt 9): 2349-2355.

77. Smith AW, Skilling DE, Ensley PK, Benirschke K, Lester TL (1983) Calicivirus isolation and persistence in a pygmy chimpanzee (Pan paniscus). Science 221: 79-81.
