# Supplementary material for: Feature Selection Methods for Identifying Genetic Determinants of Host Species in RNA Viruses
Source: PLoS Comput Biol. 2013 Oct 10;9(10):e1003254. doi: 10.1371/journal.pcbi.1003254 (PMC3794897; doi:10.1371/journal.pcbi.1003254)
Supplement: Table S3 — SARS coronavirus sequences used. (DOCX) [file pcbi.1003254.s008.docx]

**Table S3. SARS coronavirus sequences used.**

| Sequence ID | Class | Genbank reference |
| --- | --- | --- |
| SZ3 | PC03 | AY304486 |
| SZ16 | PC03 | AY304488 |
| PC4-13 | PC04 | AY613948 |
| PC4-136 | PC04 | AY613949 |
| PC4-227 | PC04 | AY613950 |
| GZ02 | HP03E | AY390556 |
| HGZ8L1-A | HP03E | AY394981 |
| ZS-C | HP03E | AY395003 |
| ZS-B | HP03E | AY394996 |
| ZS-A | HP03E | AY394997 |
| HSZ-A | HP03E | AY394984 |
| HSZ-Cc | HP03E | AY394995 |
| HSZ-Cb | HP03E | AY394986 |
| HSZ-Bc | HP03E | AY394994 |
| HGZ8L1-B | HP03E | AY394982 |
| GZ50 | HP03M | AY304495 |
| GZ-A | HP03M | AY394977 |
| JMD | HP03M | AY394988 |
| BJ03 | HP03M | AY278490 |
| BJ02 | HP03M | AY278487 |
| BJ01 | HP03M | AY278488 |
| CUHK-W1 | HP03M | AY278554 |
| HZS2-Bb | HP03M | AY395004 |
| HZS2-A | HP03M | AY394983 |
| HZS2-D | HP03M | AY394989 |
| HZS2-E | HP03M | AY394990 |
| HZS2-C | HP03M | AY394992 |
| HZS2-Fb | HP03M | AY394987 |
| BJ04 | HP03M | AY279354 |
| NS-1 | HP03M | AY508724 |
| FRA | HP03L | AY310120 |
| SoD | HP03L | AY461660 |
| ShanghaiQXC1 | HP03L | AY463059 |
| GZ-B | HP03L | AY394978 |
| GZ-D | HP03L | AY394980 |
| GZ-C | HP03L | AY394979 |
| TOR2 | HP03L | AY274119 |
| TC1 | HP03L | AY338174 |
| TC2 | HP03L | AY338175 |
| TC3 | HP03L | AY348314 |
| URBANI | HP03L | AY278741 |
| AS | HP03L | AY427439 |
| WHU | HP03L | AY394850 |
| CUHK-SU10 | HP03L | AY282752 |
| FRANKFURT | HP03L | AY291315 |
| HKU-39849 | HP03L | AY278491 |
| A11S | HP03L | AY345986 |
| A7N | HP03L | AY345987 |
| TW1 | HP03L | AY291451 |
| TW2 | HP03L | AY502925 |
| TW3 | HP03L | AY502926 |
| TW11 | HP03L | AY502924 |
| TWC | HP03L | AY321118 |
| TWH | HP03L | AP006557 |
| GD69 | HP03L | AY313906 |
| HSR | HP03L | AY323977 |
| Sino1-11 | HP03L | AY485277 |
| Sino3-11 | HP03L | AY485278 |
| Sin850 | HP03L | AY559096 |
| Sin849 | HP03L | AY559086 |
| Sin852 | HP03L | AY559082 |
| SIN2774 | HP03L | AY283798 |
| SIN2748 | HP03L | AY283797 |
| Sin3765V | HP03L | AY559084 |
| PUMC01 | HP03L | AY350750 |
| PUMC02 | HP03L | AY357075 |
| SIN2679 | HP03L | AY283796 |
| CUHK-LC1 | HP03L | AY394998 |
| CUHK-LC2 | HP03L | AY394999 |
| CUHK-LC3 | HP03L | AY395000 |
| SZ1 | PC03 | AY304489 |
| SZ13 | PC03 | AY304487 |
| PC4-145 | PC04 | AY627046 |
| PC4-115 | PC04 | AY627044 |
| PC4-241 | PC04 | AY627048 |
| PC4-127 | PC04 | AY613951 |
| HK3-9 | Bats | GQ153544 |
| HKU3-13 | Bats | CQ153548 |
| HKU3-12 | Bats | CQ153547 |
| HKU3-6 | Bats | CQ153541 |
| HKU3-1 | Bats | DQ22305 |
| GZ0401 | HP04 | AY568539 |
| GZ0402 | HP04 | AY613947 |
| GZ0403 | HP04 | AY613953 |
| GD03T0013 | HP04 | AY525636 |
| HKU-39849 | HP04 | AY278491 |
