# Supplementary material for: Feature Selection Methods for Identifying Genetic Determinants of Host Species in RNA Viruses
Source: PLoS Comput Biol. 2013 Oct 10;9(10):e1003254. doi: 10.1371/journal.pcbi.1003254 (PMC3794897; doi:10.1371/journal.pcbi.1003254)
Supplement: Table S4 — SARS-like virus nucleotide variants present in the feature selected alleles and corresponding amino acid residues. Putative residue positions in the 3D conformation of the spike protein were suggested by [22]. Synonymous substitutions are shown in italic. (DOCX) [file pcbi.1003254.s009.docx]

**Table S4.** SARS-like virus nucleotide variants present in the feature selected alleles and corresponding amino acid residues. Putative residue positions in the 3D conformation of the spike protein were suggested by [[22](#_ENREF_22)]. Synonymous substitutions are shown in italic.

| AA residue | Single-nucleotide variants | AA switch | 3D conformation |
| --- | --- | --- | --- |
| 77 | **ag** | **D-G** | **Exposed top** |
| 239 | **tc** | **L-S** | **Partially exposed side** |
| 244 | **ct** | **T-I** | **Buried** |
| 311 | **ga** | **G-R** | **Exposed side** |
| *342* | *ga* | *R-R* | *Exposed top* |
| 344 | **ga** | **R-K** | **Partially exposed side** |
| 360 | **tc** | **F-S** | **Exposed top** |
| *461* | *ct* | *S-S* | *Exposed side* |
| 472 | **tc** | **L-P** | **Exposed side** |
| 479 | **ta/ag** | **N,R,K** | **Exposed side** |
| 487 | **cg** | **T-S** | **Exposed side** |
| 665 | **tc** | **L-S** | **Buried** |
| 778 | **gt** | **D-Y** | **Buried** |
| *1025* | *ct* | *C-C* | *Exposed side* |
| 1163 | **ga** | **E-K** | **Buried** |
