# Supplementary material for: Feature Selection Methods for Identifying Genetic Determinants of Host Species in RNA Viruses
Source: PLoS Comput Biol. 2013 Oct 10;9(10):e1003254. doi: 10.1371/journal.pcbi.1003254 (PMC3794897; doi:10.1371/journal.pcbi.1003254)
Supplement: Table S5 — Rabies virus nucleoprotein sequences analysed. (DOCX) [file pcbi.1003254.s010.docx]

Table S5. Rabies virus nucleoprotein sequences analysed.

| Sequence ID | Bat species where the virus was found | Genbank reference |
| --- | --- | --- |
| AZ1968 | *E.fuscus* | GU644642 |
| CA237 | *E.fuscus* | GU644644 |
| CA0120 | *E.fuscus* | GU644647 |
| CA148 | *E.fuscus* | GU644649 |
| GA31940 | *E.fuscus* | GU644652 |
| GA36568 | *E.fuscus* | GU644653 |
| IA381 | *E.fuscus* | GU644655 |
| IA543 | *E.fuscus* | GU644656 |
| MI1271 | *E.fuscus* | GU644658 |
| MI1586 | *E.fuscus* | GU644660 |
| MI1833 | *E.fuscus* | GU644662 |
| MI1865 | *E.fuscus* | GU644667 |
| MI596 | *E.fuscus* | GU644670 |
| NJ1049 | *E.fuscus* | GU644672 |
| NJ511 | *E.fuscus* | GU644674 |
| NJ949 | *E.fuscus* | GU644675 |
| VA2057 | *E.fuscus* | GU644677 |
| WA369 | *E.fuscus* | GU644679 |
| WA173 | *E.fuscus* | GU644681 |
| WA1159 | *E.fuscus* | GU644685 |
| WA1455 | *E.fuscus* | GU644687 |
| WA1625 | *E.fuscus* | GU644690 |
| WA2017 | *E.fuscus* | GU644695 |
| MI738 | *E.fuscus* | GU644819 |
| ID7318 | *E.fuscus* | GU644801 |
| GA32657 | *L.borealis* | GU644856 |
| TN272 | *L.borealis* | GU644859 |
| VA1924 | *L.borealis* | GU644877 |
| VA1198 | *L.borealis* | GU644879 |
| TX5943 | *L.borealis* | GU644863 |
| TX6151 | *L.borealis* | GU644865 |
| GA60243 | *L.borealis* | GU644700 |
| TN33 | *L.borealis* | GU644705 |
| TX2356 | *L.borealis* | GU644708 |
| TX5976 | *L.borealis* | GU644711 |
| FL701 | *L.borealis* | GU644698 |
| AZ1838 | *L.cinereus* | GU644712 |
| AZ7771 | *L.cinereus* | GU644714 |
| CA030 | *L.cinereus* | GU644885 |
| CA3872 | *L.cinereus* | GU644889 |
| CA7979 | *L.cinereus* | GU644894 |
| IN669 | *L.cinereus* | GU644900 |
| TN898 | *L.cinereus* | GU644903 |
| TX6464 | *L.cinereus* | GU644906 |
| ID7227 | *L.cinereus* | GU644715 |
| TN183 | *L.cinereus* | GU644717 |
| WA2085 | *L.cinereus* | GU644721 |
| FL769 | *L.seminolus* | GU644731 |
| GA7034 | *L.seminolus* | GU644732 |
| FL732 | *L.seminolus* | GU644939 |
| FL792 | *L.seminolus* | GU644941 |
| FL942 | *L.seminolus* | GU644943 |
| TX6265 | *L.seminolus* | GU644945 |
| TX5419 | *L.seminolus* | GU644944 |
| TX5512 | *L.seminolus* | GU644735 |
| TX6127 | *L.seminolus* | GU644737 |
| TX6197 | *L.seminolus* | GU644733 |
| AZ2405 | *T.brasiliensis* | GU644760 |
| AZ3086 | *T.brasiliensis* | GU644762 |
| CA0052 | *T.brasiliensis* | GU644766 |
| CA1984 | *T.brasiliensis* | GU644770 |
| CA46 | *T.brasiliensis* | GU644774 |
| FL148 | *T.brasiliensis* | GU644777 |
| GA135 | *T.brasiliensis* | GU644780 |
| MS076 | *T.brasiliensis* | GU644782 |
| TX5775 | *T.brasiliensis* | GU644784 |
| CA0057 | *T.brasiliensis* | GU644992 |
| FL1318 | *T.brasiliensis* | GU644994 |
| TX5638 | *T.brasiliensis* | GU645003 |
| TX5218 | *T.brasiliensis* | GU645007 |
