# Supplementary material for: Feature Selection Methods for Identifying Genetic Determinants of Host Species in RNA Viruses
Source: PLoS Comput Biol. 2013 Oct 10;9(10):e1003254. doi: 10.1371/journal.pcbi.1003254 (PMC3794897; doi:10.1371/journal.pcbi.1003254)
Supplement: Table S6 — Random forest host reservoir prediction probabilities for rabies viruses, excluding the putative species transition samples. (DOCX) [file pcbi.1003254.s011.docx]

Table S6. Random forest host reservoir prediction probabilities for rabies viruses, excluding the putative species transition samples.
