# Supplementary material for: Feature Selection Methods for Identifying Genetic Determinants of Host Species in RNA Viruses
Source: PLoS Comput Biol. 2013 Oct 10;9(10):e1003254. doi: 10.1371/journal.pcbi.1003254 (PMC3794897; doi:10.1371/journal.pcbi.1003254)
Supplement: Table S7 — Positions selected as host-specific in the influenza A HA analysis. The positions are ordered according to their predicted RFA importance for classification. (DOCX) [file pcbi.1003254.s012.docx]

Table S7. Positions selected as host-specific in the influenza A HA analysis. The positions are ordered according to their predicted RFA importance for classification.

| Position | In receptor binding domain? | Antigenic site |
| --- | --- | --- |
| 171 | YES | Ca antigenic site |
| 317 | *NO* |  |
| 305 | *NO* |  |
| 242 | YES |  |
| 145 | YES | Ca antigenic site |
| 263 | YES |  |
| 80 | *NO* | Cb antigenic site |
| 369 | *NO* |  |
| 244 | YES |  |
| 264 | YES |  |
| 377 | *NO* |  |
| 261 | YES |  |
| 531 | *NO* |  |
| 199 | YES |  |
| 275 | YES |  |
| 155 | YES |  |
| 46 | *NO* |  |
| 271 | *NO* |  |
| 159 | YES | Sa antigenic site |
| 198 | YES | Sb antigenic site |
| 187 | YES | Sb antigenic site |
| 189 | YES | Sb antigenic site |
| 219 | YES |  |
| 92 | *NO* |  |
| 286 | *NO* |  |
| 55 | *NO* |  |
| 81 | *NO* | Cb antigenic site |
| 196 | YES | Sb antigenic site |
| 377 | *NO* |  |
| 208 | YES | Ca antigenic site |
| 324 | *NO* |  |
| 156 | YES | Sa antigenic site |
| 248 | YES |  |
| 348 | *NO* |  |
| 131 | YES |  |
| 403 | *NO* |  |
| 149 | YES |  |
| 324 | *NO* |  |
| 123 | *NO* |  |
| 91 | *NO* |  |
| 188 | YES | Sb antigenic site |
| 205 | YES |  |
| 497 | *NO* |  |
| 169 | YES | Ca antigenic site |
| 227 | YES |  |
| 301 | *NO* |  |
| 476 | *NO* |  |
| 318 | *NO* |  |
| 158 | YES | Sa antigenic site |
