# Supplementary material for: Feature Selection Methods for Identifying Genetic Determinants of Host Species in RNA Viruses
Source: PLoS Comput Biol. 2013 Oct 10;9(10):e1003254. doi: 10.1371/journal.pcbi.1003254 (PMC3794897; doi:10.1371/journal.pcbi.1003254)
Supplement: Table S8 — Sequences used for the analysis of the influenza A PB2 segment by subtype and host reservoir. (DOCX) [file pcbi.1003254.s013.docx]

Table S8. Sequences used for the analysis of the influenza A PB2 segment by subtype and host reservoir.

| **Influenza subtype** | **Host** | **Genbank reference** |
| --- | --- | --- |
| **H1N1** | *Human* | AEX92937, AEC47217, ADJ40430, ABD60965, ABO38075, ABD60965, ACF41943, AEX33956, AEX34778, AEX33934, AEX33364, AEX33386, AET84324, AET84346, ADX99875, ACO94869, ADT78915, ACJ25130, AEX92937, ACO94647, ABD59805, ABD15525, ABC42760, ABP49491, ABP49458, ABA55038, ADJ40430, AEX33868, AEN02502, ACV49544, AEA04364, ADY04723, ACN32522, ADC45622, ABF21236, ABM67061, ABD79111, ABF21235, ABD61745, ABY51082, ACK99475, ADX99875, ABC86247, ABY51115, ABN51076, ABO44133, ABD60866, ABI20847, AAF99675, AAF99674 |
|  | *Avian* | ABB20386, ADU17047, ADT79360, ADM26555, ACJ14455, ABB19550, ABB19539, ACZ45274, ABG88266, ADE75022, ACI41088, ACF25468, ABB19627, AEK70453, ADU53624, ACG59852, ABS70336, ABB17160, ACJ14477, ACD88712, AET75555, ADQ93374, ACF25038, ABB88032, ADH96314, ABB19638, ABG88266, ABG88222, ADR00811, ACI41088, ACT84996, AET84466, ACY79828, AET11796, ADN78167, ACZ45593, ACI40999, ABB19584, ABB19429, ABM21970, ABG88266, ACJ14455, ACJ14488, ACD88712, ACF25617, ACF25607 |
|  | *Swine* | AEO91800, AEO91789, AEO91688, AEO91677, BAH02157, BAH02047, ADY70593, ABE27163, ABD95721, AAA43652, BAH02077, BAH02067, ABB86930, ABY81436, ABY84693, ABX58656, ABW86595, ABW71491, ABW71530, ABR28668, ABU80419, ABQ45468, ABD95721, ABD78114, AAU25842, ACN39286, AAA43126, ACD85164, AEO91942, AEN94827, AEN94838, AEN94860, ADY71467, ACY67073, ACY67068, ACA25347, ADF83498, ABW38020, ACD65212, ACK57723, ACT84274, ACM17261, ACQ84506, ACR84043, ACE78126, BAH02147 |
| **H1N2** | *Human* | AAZ74384, ABB53613, ABB03144, AAY78948, ABB83036, ABB83025, ABB53739, ABB53613, ABB04982, ABB03144, ABB03111, ABA42290, ABA08518, ABA06552, ABA06520, AAZ79559, AAZ79548, AAZ74384, AAY78948, ABK40005, ABD94953 |
|  | *Avian* | ADP07173, ADP07184, AAO65605, AEM76009, AEK50528, AEM75844, ABI84865 |
|  | *Swine* | AEO91822, AEO91756, AEN94816, ACR39492, AEN94849, ACV42047, ACV42036, ACV42113, ACV42058, ACM17250, ACI89695, ABB86920, ABB86910, ABB86890, AAL87936, AAL87935, AAL87934, AAL87933, AAL87932, AAL87931, AAL87930, AAL87929, AAF76002, ADU85822 |
| **H2N2** | *Human* | ACV49599, ACD56312, ACV49588, ACD56301, AAA43595, ABQ44470, ABQ44448, ABQ01365, ABP49480, ABP49469, ABO52312, ABO52257, ABO44111, ABO44100, ABO44067, ABO38317, ABO38306, AAO46265, AAO46263, AAO46261, AAO46262, AAO46260, AAO46255, AAO46252, AAO46267 |
|  | *Avian* | ACD88680, ACZ45277, ACZ45276, ACZ45604, ABI84765, ACJ69306, ABB20239, ADK61179, AAY87429, |
| **H3N2** | *Human* | ABA43346, AEG65790, AAY18599, AAZ38527, ABA16488, ABA12787, AAZ38527, ABY51236, ABW91657, ABW91558, Q6XTL0, ABD61767, ABN51109, ABW81537, AEW31152, AEK28923, AEI54500, AEI54355, AEH77285, ADK98919, AEG64965, AEG64930, AEG64899, ADI75938, AAA43131, AAY28627, ACO94759, AAB05770, ACO36649, ABD16592, ABD17344, ABC86147, ABC67871, AAZ38560, ABC54678, ABC46586, ABB96351, AAY98085, ABY81381, ABY51280, ABV30458, ABO52334, ABQ01376, AAO46559, AAO46553, AAZ43393, AAZ80018 |
|  | *Avian* | AET74605, AET75471, ABB87387, ABL75573, AEM75712, ABS89408, ACE76845,ACF25011, ABB19736, ACF25227, ABO76956, ABK80024, ABI84783, AET75517, AET78151, ACF25564, ABI84783, ACV41547, ACE76845, ACS92936, AEM75533, AEM75910, ABI48016, AEM75227, ADG85837, ACX55520, ACE76847, ACF25004, ABR37494, ABO51839, ACX55509, ACZ45325, AEP95316, ABC59716, ABQ41885, ACF25539, ACT84513, ACF25553, ACD88658, ABF18007, ACD88669, ACN86434 |
|  | *Swine* | AET50906, AEL31736, AEY75646, AEY75635, AEZ01206, AEZ01206, AEO91898, ADZ05631, ABF18006, ABF18005, AAG01748, ABD61561, AEZ02270, AEZ02136, AEZ02119, AEZ01204, AEY75615, AEL31736, AEO91920, AEO91876, AEO91723, AEO91620, AFD32623, ADV58918, ADV58923, ADX60669, ADU85679, ACY67067, ACY67066, ABQ41895, ADZ05634, ACS92891, ADZ05633, ACF04401, ABF18004, ABF18003, ABD61259 |
| **H5N1** | *Human* | ABX57880, AEO89177, AEO89168, AEO89159, AEO89150, AEO89114, AEO89078, AEO89035, AEO89026, AEO89001, AEO88960, AEA50981, ACU46651, ACS93349, ABI36432, ABI36211, ABF01751, AAF74313, AAF74312 |
|  | *Avian* | ACZ36526, ACZ45409, ACZ36528, ACF36751, ADA00384, ADQ92592, ACZ36492, ACU15977, ACV41646, AET74617, ABI84616, AEM75723, AEB66743, AEK70482, AEK70730, ACZ45342, ADA82210, ACZ36525, ACZ45416, ABQ43786, ACH43177, ABQ12376 |
| **H3N8** | *Avian* | AET74594, AET77400, AET75482, AET74686, AET75566, AET77426, ADP07206, ADP06931, AET76376, AET78129, ADU16992, ADP13362, ACZ45454, ACD88647, ACF33771, AET75300, ACI90165, ACE76621, ABB87799, ABO76923, ABI95484, ABI92279, AET75392, ACI90165, ADP07035, ADA82155, ACV89748, ADU53697, ABO52136, ABL67851, AET74845, ABI84421, ABI47980, AET74823, AET74856, AEB39752, ADU16509, ADG85844, ABO51894, ABI92268, ABI84947, ACZ45455, ACZ36533, ACN86602, ABL67142, ACS68444 |
|  | *Canine* | Top of Form  ADM29690, ADM29679, ADM29657, ADM29668, ADM29646, ADM29613, ADM29602, ADM29591, ADM29580, ADM29569, ADM29547, ADM29536, ADM29525, ADM29514, ADM29503, ADM29492, ADM29481, ADM29470, ADM29459, ADM29448, ADM29437, ADM29437, ADM29415, ADM29404, ADM29393, ADM29382, ADM29371, ADM29360, ADM29349, ADM29327, ADM29316, ADM29338, ADM29239, ACD62542, ACD62534  Bottom of Form |
|  | *Equine* | Top of Form  AEM60123, AEM60134, ADM29635, ADM29624, ADM29558, ADM29305, ADM29272, ACZ45460, ABY81491, ACD85175, ACZ45401, ACA24666, ACA24578, ACF22125, ACD85186, ACD56145, ACD85296, ACA96821, ACA96810, ACA96551, ACA24688, ACA24677, ACA24644, ACA24926, ACA24655, ACA24633, ACA24622, ACA24567, ACA24545, AAA43133, ACA24556, ABB17182, ABY81612, ABY81601, ABY81590, ABY81579, ABY81524, ABY81513, ABY81502, ABY81480, ABY81469, ABY81458, ABM21948, AAX23572Bottom of Form |
| **H7N7** | *Avian* | Top of Form  ACS68434, ABB87843, ABB87832, ABI84989, ADU17607, ADU20316, ADP07503, ABI94590, ACP43601, ABI94590, AET50895, ABI85083, ADN65122Bottom of Form |
|  | *Equine* | Top of Form  ACQ73421, ACL12095, ACB30161, AAA43141, AEM60146, ACL12106, ACL12117, ACL12128, AEA04407, ACQ73421Bottom of Form |
