# Supplementary material for: Feature Selection Methods for Identifying Genetic Determinants of Host Species in RNA Viruses
Source: PLoS Comput Biol. 2013 Oct 10;9(10):e1003254. doi: 10.1371/journal.pcbi.1003254 (PMC3794897; doi:10.1371/journal.pcbi.1003254)
Supplement: Table S9 — Influenza virus host reservoir relevant amino acids and their respective level of conservation across all viral subtypes (δh, δa, δs, δc, and δe). (DOCX) [file pcbi.1003254.s014.docx]

**Table S9.** Influenza virus host reservoir relevant amino acids and their respective level of conservation across all viral subtypes (δh, δa, δs, δc, and δe).

|  | Human | δh | Avian | δa | Swine | δs | Canine | δc | Equine | δe |
| --- | --- | --- | --- | --- | --- | --- | --- | --- | --- | --- |
| **64** | T | 0.89 | M | 0.98 | M | 0.71 | M | 1 | M | 0.94 |
| **65** | E | 0.98 | E | 0.94 | D | 0.61 | E | 1 | E | 0.96 |
| **81** | M | 0.66 | T | 1 | T | 0.89 | T | 1 | T | 0.94 |
| **105** | V | 0.61 | T | 0.98 | T | 0.93 | T | 1 | T | 1 |
| **147** | I | 0.92 | I | 0.9 | T | 0.63 | V | 1 | V | 0.79 |
| **199** | S | 0.88 | A | 0.97 | A | 0.72 | A | 1 | A | 1 |
| **271** | A | 0.86 | T | 0.93 | A | 0.68 | T | 1 | T | 0.98 |
| **292** | T | 0.82 | I | 0.94 | I | 0.78 | T | 1 | I | 0.98 |
| **299** | R | 0.99 | R | 0.97 | R | 0.76 | K | 1 | K | 0.96 |
| **368** | K | 0.81 | R | 0.98 | R | 0.7 | R | 1 | R | 0.96 |
| **475** | M | 0.88 | L | 0.97 | L | 0.72 | L | 1 | L | 1 |
| **530** | T | 1 | T | 0.99 | T | 1 | I | 1 | I | 0.94 |
| **559** | T | 0.68 | T | 0.9 | - | 0.52 | N | 0.66 | I | 0.94 |
| **567** | N | 0.87 | D | 1 | D | 0.94 | D | 1 | D | 1 |
| **586** | K | 1 | K | 0.98 | K | 0.97 | R | 1 | R | 0.94 |
| **588** | I | 0.85 | A | 0.92 | - | 0.56 | T | 1 | T | 0.94 |
| **591** | Q | 0.99 | Q | 0.95 | R | 0.61 | Q | 1 | Q | 0.98 |
| **613** | T | 0.76 | V | 0.99 | V | 0.91 | A | 1 | A | 0.92 |
| **627** | K | 0.94 | E | 0.98 | E | 0.71 | E | 1 | E | 1 |
| **661** | T | 0.83 | A | 0.97 | A | 0.68 | A | 0.66 | A | 0.96 |
| **674** | T | 0.85 | A | 1 | A | 0.87 | A | 1 | A | 1 |
| **701** | D | 0.99 | D | 0.99 | D | 0.92 | N | 1 | N | 0.98 |
| **702** | R | 0.85 | K | 0.99 | K | 0.9 | K | 1 | K | 1 |
|  |  |  |  |  |  |  |  |  |  |  |
|  |  | Small non polar | | | |  | Polar positive | | | |
|  |  | Small non polar aliphatic/aromatic | | | |  | Polar neutral/negative | | | |
|  |  | Hydrophobic non polar aliphatic | | | | | | | | |
