# Supplementary material for: Feature Selection Methods for Identifying Genetic Determinants of Host Species in RNA Viruses
Source: PLoS Comput Biol. 2013 Oct 10;9(10):e1003254. doi: 10.1371/journal.pcbi.1003254 (PMC3794897; doi:10.1371/journal.pcbi.1003254)
Supplement: Table S10 — Mean pairwise distance (measured in terms of amino acid differences) between and within host reservoir groups for the influenza viruses used in the PB2 analysis. (DOCX) [file pcbi.1003254.s015.docx]

Table S10. Mean pairwise distance (measured in terms of amino acid differences) between and within host reservoir groups for the influenza viruses used in the PB2 analysis.

|  | Human | Avian | Swine | Canine | Equine |
| --- | --- | --- | --- | --- | --- |
| Human | **25.406** |  |  |  |  |
| Avian | 32.822 | **8.3864** |  |  |  |
| Swine | 42.005 | 24.37 | **30.982** |  |  |
| Canine | 45.606 | 25.288 | 37.911 | **3.2975** |  |
| Equine | 43.331 | 20.884 | 34.379 | 13.66 | **11.399** |
