# Supplementary material for: Feature Selection Methods for Identifying Genetic Determinants of Host Species in RNA Viruses
Source: PLoS Comput Biol. 2013 Oct 10;9(10):e1003254. doi: 10.1371/journal.pcbi.1003254 (PMC3794897; doi:10.1371/journal.pcbi.1003254)
Supplement: Table S11 — Summary of the classification type random forest algorithms performed and overall prediction error. (DOCX) [file pcbi.1003254.s016.docx]

| **Viruses** | **Genome region** | **# viruses** | **Sequence size (# alleles)** | **# selected alleles (# positions)** | **Out-of-bag prediction error** |
| --- | --- | --- | --- | --- | --- |
| Flavivirus | Polymerase | 24 | 270 (831) | 8 (8) | 0.167 |
| Alphavirus | Polymerase | 14 | 309 (319) | 11 (9) | 0.077 |
| Paramyxoviridae | Polymerase | 29 | 2386 (16404) | 9 (9) | 0.138 |
| Caliciviridae | Polymerase | 28 | 183 (684) | 10 (9) | 0.444 |
| Rabies | Nucleoprotein | 70 | 594 (397) | 18 (12) | 0.086 |
| SARS | Spike | 85 | 4124 (4673) | 22 (15) | 0.059 |
| Influenza A | Haemagglutinin (HA) | 1908 | 567 (1200) | 66 (48) | 0.014 |
|  | Polymerase (PB2) | 557 | 755 (464) | 48 (23) | 0.052 |

Table S11. Summary of the classification type random forest algorithms performed and overall prediction error.
