# Supplementary material for: Feature Selection Methods for Identifying Genetic Determinants of Host Species in RNA Viruses
Source: PLoS Comput Biol. 2013 Oct 10;9(10):e1003254. doi: 10.1371/journal.pcbi.1003254 (PMC3794897; doi:10.1371/journal.pcbi.1003254)
Supplement: Text S1 — Includes a more detailed description of the methods used throughout, as well as Figures S1, S2, S3, S4, S5 and Tables S1, S2, S3, S4, S5, S6, S7, S8, S9, S10, S11. (DOCX) [file pcbi.1003254.s017.docx]

# Supporting Information

# Materials and Methods

# Analysing RNA viral sequences with the Random Forest Algorithm

Despite much progress, the quest for understanding the drivers of emergence of new pathogens has yet to provide quantitative means to assess the threat of emergence posed by new viral variants. The wealth of pathogen sequence data becoming available makes identification of pathogen genomic markers of emergence one of the more promising approaches [[1](#_ENREF_1)], particularly for RNA viruses given their high mutation rate and resulting high diversity at the population level [[2](#_ENREF_2)].

Over the past decade, sophisticated statistical techniques enabling the discrimination of phenotype relevant variables (disease phenotype, gene expression pattern) have become more prominent in biomedical research. One such approach is feature selection, which attempts to find the subset of relevant features that can inform a classification algorithm accurately [[3](#_ENREF_3)]. Here we focus on the random forest algorithm (RFA) feature selection method. In the context of viral sequence analysis the Random Forest Algorithm (RFA) can be used as an approach to identify genetic information that allows reliable classification of RNA viruses by reservoir host species. In doing so we expect to uncover host specific amino acids or nucleotides against which new sequences can be compared, in order to predict the probability of those viruses being adapted to each of its known host reservoirs, and thus use it as a tool for the assessment of zoonotic disease emergence potential.

Kimura’s neutral theory of molecular evolution postulates that the vast majority of evolutionary changes are caused by random drift of selectively neutral mutants [[4](#_ENREF_4)]. Changes that enable a given pathogen to cross the species barrier might be neutral (or deleterious) in the “donor” host species, but are inevitably subject to strong selective pressures in the recipient host species. We would then expect the mutants under neutral and purifying selection in the donor species to have a different frequency when compared to the same mutants in the recipient hosts. Of particular interest is the impact of selection operating on the recipient host species. Due to tissue tropism, host compatibility and transmission potential constraints, selection pressure should be intense and the number of successful mutants quite limited. If specific hosts impose specific evolutionary landscapes on pathogens, that should translate into host-specific signature genetic markers. Hence, comparisons of allele frequencies between sequences of pathogens from humans and rodents, for instance, should reveal a large subset of non-discriminant alleles between human and rodent samples and a smaller subset of human and rodent specific alleles.

We chose to use an aggressive variable selection method in line with the idea that purifying selection is the main driver of allele frequency variance across hosts [[5](#_ENREF_5),[6](#_ENREF_6)]. The great majority of amino acid variation in natural populations of RNA viruses is due to transient deleterious mutations, later purged by purifying selection, and thus limiting virus adaptability to new hosts [[5](#_ENREF_5)]; This is supported by the extent to which host phylogeny constrains cross-species transitions [[7](#_ENREF_7),[8](#_ENREF_8),[9](#_ENREF_9),[10](#_ENREF_10)]. Such purifying selection appears to be more intense in RNA viruses than DNA viruses, though present in both [[6](#_ENREF_6)]. These selective pressures are in fact so forceful that only a few key single point mutations can be sufficient to permit cross-species infection (e.g. coronaviruses [[11](#_ENREF_11)] and influenza [[12](#_ENREF_12)]). Influenza’s PB2 627 mutation is a good example of this: whereas the amino acid at position 627 in almost every avian influenza A virus is glutamic acid, in human influenza A viruses it is typically a lysine. RFA variable selection is tailored to identify subsets of host specific alleles whilst providing a measure of allelic importance for classification (implicitly accounting for possible allelic interactions), and extrapolating a probability of host origin for each virus.

## Caveats and potential sources of bias

### Overfitting

The prediction error of the RFA is calculated by the ‘.632+’ bootstrap method which relies on the relationship between the resubstitution error and the misclassification error on samples not used to train the algorithm (so-called ‘out-of-bag’ [OOB] samples) [[13](#_ENREF_13),[14](#_ENREF_14)]. The resubstitution error essentially measures the proportion of observations in the original dataset that are misclassified by the decision trees within the random forest. Variable importance is also estimated using the samples which are left out of the training set at each split of the tree, making the random forest algorithm robust to over-fitting. The original description of the method provides a demonstration of how Random Forests converge, explaining why they do not over-fit as the number of trees grows to infinity, instead producing a limiting value of the prediction error [[15](#_ENREF_15)].

### Sampling bias and founder effects

It is important to be aware of how inhomogeneous sampling of viruses (both in space and time) can affect classification and feature selection. RFA accuracy is only as good as how well the variance in allelic frequencies in the training set represents true differences between allele frequencies observed between hosts. If known biases in sampling are present in training, RFA can be easily adjusted to assign different weights to different sets of data [[16](#_ENREF_16),[17](#_ENREF_17),[18](#_ENREF_18)], such that unevenly represented groups have equal impact on the overall prediction error. Also, variable selection at each node of the classification tree can be undertaken with replacement, which has been shown to decrease the overestimation of the true classification error [[17](#_ENREF_17)]. We stress that RFA is an ensemble method in which classification is performed by voting of multiple unbiased weak classifiers (decision trees) which use random samples of the original data [[15](#_ENREF_15)], and in which only a random sample of all the variables is chosen for each tree. The result is multiple low-correlation trees each assessing the importance of a subset of variables to the classification of a subset of the data. Combining the ensemble of trees results in a low bias and low variance algorithm [[14](#_ENREF_14),[19](#_ENREF_19)]. Cross-validation of the results further reduces any remaining sources of bias.

Founder effects can be particularly challenging to detect in allelic frequency analyses across populations [[20](#_ENREF_20)]. In the context of zoonotic disease emergence and cross species transition events, founder effects occur when there is a fairly diverse viral population in their donor species and a collapse of said diversity when/if the virus infects a new species. However, as mentioned above, most amino acid variation in natural populations of RNA viruses is essentially comprised of transient deleterious mutations which are purged by purifying selection. Whilst founder effects can be extremely significant in the variations observed in a given host species, they is much less likely to be a major factor when dealing with differences between different hosts, due to extremely restrictive host barriers [[21](#_ENREF_21)]. One cannot discard the possibility of founder effects interference though, so we have performed a functional analysis to assess whether RFA identifies amino acids with potential functional relevance to host specificity and which might be expected to be functionally constrained within a single host species. Amino acids on the surface of proteins potentially interacting directly with host molecules are typically not highly malleable, meaning any mutations are likely to be strongly deleterious. If changes in these amino acids are observed, they are therefore more likely to be significant for host adaptation. We must recognise, however, that although we are able to identify clear host specific markers that appear to have functional relevance, the exact amino acids/allelic frequencies seen at some sites might arise due to drift since the cross species transition, and not necessarily imputable to host adaptation.

### Other sources of bias

Standard genetic sequences, with each position coded by a character indicating a nucleotide or amino acid, should not be used directly on the decision trees composing the random forest since there is a bias associated with having category number variation across the sequences (13). Whilst this effect would be marginal when dealing with nucleotide sequences (given there are only 4 categories), it can become an issue with amino acid sequences where the sites presenting with the highest number of polymorphisms would be tend to be assigned a higher level of significance. We therefore recoded the information within a given sequence alignment into an allele frequency matrix, using the adegenet R package [12]. This recoding is illustrated by Table S1. Starting from a multiple sequence alignment, all conserved sites are discarded, and a presence/absence matrix of all other alleles is assembled. Since we are dealing with RNA viruses, this matrix is actually equivalent to a presence/absence matrix of amino acid/nucleotide types in polymorphic sites.

## Feature Selection

Feature selection aims at selecting a subset of relevant features for building robust learning models, by reducing the number of features, through extraction of irrelevant, redundant, or noisy data. In biology, it is mainly applied in the analysis of microarray data and in genome wide association studies, particularly for identification of markers for disease risk and drug resistance [[22](#_ENREF_22),[23](#_ENREF_23),[24](#_ENREF_24)]. As an embedded feature selection technique, the random forest algorithm has several advantages over other types of algorithm. Embedded methods incorporate variable selection as part of the training process, thus, directly linking variable importance to the learner used to model the outcome. They are usually more effective than other methods because they make integral use of the training data (whereas other methods require a split into training and validation sets) and incorporate feature selection as a part of the model training process [[25](#_ENREF_25)]. In a previous study, a heuristic version of an embedded feature selection algorithm found several mutation combinations associated with viral host specificity and virulence [[26](#_ENREF_26)].

The importance measure of each variable is obtained as the loss of accuracy of classification caused by the random permutation of attribute values for that variable. In practical terms, the algorithm takes as inputs *n* sequences of size *p* (*p* being the number of unique alleles across the sequence in this case), constituting a matrix similar to one represented by Table S1B, and a class variable *h* defining a specific host reservoir for each sequence. The algorithm then tries to find a subset $s_{p}\subset p$of alleles whose contribution to the explanation of the class variable is significantly greater than that of random permutations of the values of variables $s_{p}$ in the out-of-bag samples [[15](#_ENREF_15)].

All the random forest algorithm runs were performed with the *varSelRF* R package [[27](#_ENREF_27)], using 2000 trees, with $\sqrt{2p}$variables evaluated at each split of those trees.

### Variable selection using RFA

Variable selection is performed by removing a fixed fraction of variables from one random forest to the next in all instances of an iterative random forest fit. The remaining subset of variables accounts for the smallest out-of-bag (OOB) error rate in each iteration. By default, the fraction of dropped variables is set to 20%, thus keeping the run time fast, and increasing the resolution as the number of variables decreases [[14](#_ENREF_14)]. Variable importances are not re-calculated at each iteration to preclude any overfitting issues [[28](#_ENREF_28)]. At the end of the iterative procedure, the solution with the smallest number of variables whose error rate is within one standard error of the minimum error rate of all forests is kept. Since variable selection is performed by minimising the OOB error rate and not simply by looking at the variable importance resulting from random permutations of the values of variables at the nodes of the trees [[27](#_ENREF_27)], selected variables might actually have a lower importance value than some unselected variables. This is precisely what happens in the SARS example, as illustrate by Figure 4 and is an indicator of allelic interactions.

### Robustness of the feature selection procedure

Even though the RFA is unlikely to over-fit, we carried out a cross-validation of the algorithm by performing multiple bootstrap runs of the feature selection procedure. Each bootstrap run is a new realisation of the complete feature selection procedure, thus removing selection bias concerns on the importance of the most significant variables. We can then make inferences on the cross-validated prediction performance of the selected random forest models with sequentially reduced number of predictors (ranked by variable importance). The solution stability can be evaluated by plotting the frequency with which the X highest ranked positions are selected as significant over a set of bootstrap runs. Figures S1 and S2 display the robustness of the results presented here and highlight how stable the solutions are when using large datasets (Figure S2).

## Principal Component Analysis

Visually representing variation in a set of genetic sequences is challenging due to the high dimensionality of the datasets, typically consisting of dozens of sequences containing hundreds or thousands of nucleotides/amino acids. Phylogenetic analysis is of course the dominant method adopted, but dimensional reduction methods such as principal component analysis (PCA) offer a less model-driven approach to illustrating the differences between a set of viral sequences [[29](#_ENREF_29),[30](#_ENREF_30),[31](#_ENREF_31),[32](#_ENREF_32),[33](#_ENREF_33),[34](#_ENREF_34),[35](#_ENREF_35)]. We gain a simple visual assessment of how well feature selection performs in phenotypic classification by applying PCA to both the original sequence dataset and the dataset consisting exclusively of variables selected by feature selection. The PCAs presented throughout this paper were performed using the *ade4* R package [[36](#_ENREF_36)]. PCA is typically used to identify patterns in high dimensional data, transforming the data in such a way as to highlight significant relationships between them. It has been extensively applied to examine relationships between genetic sequences, revealing host specificity [[35](#_ENREF_35)] and spatial distribution patterns [[31](#_ENREF_31),[32](#_ENREF_32)], for example. Essentially, the information regarding all amino acid or nucleotide differences between any two viral sequences is summarized into a score for each of the principal components. Selecting the first two principal components, which explain the largest fraction of the variance in the dataset, one can observe the relative differences between all sequences as the distances between a set of points in two dimensional space.

The basic premise of the PCA approach is to create a new set of variables, the principal components, which are a linear combination of the original variables [[37](#_ENREF_37)]. The method assures that all the principal components are orthogonal to each other, so there is no redundant information. Given a sequence alignment *X* with *p* variables (amino acid or nucleotide positions) and *n* samples (different isolates of the same species or different species), variables *p* are transformed into allele frequency variables and transformed such that they are centred on the mean frequency for each variable. Centring the data makes sure that the cloud of data points is centred on the origin of the principal components, whilst ensuring that relationships between the data and the variables are preserved. By definition, the number of principal components (*r*) cannot exceed the number of individual alleles ($r\leq min(n,p)$). The principal components are then given by a linear combination of the original data through a set of regression coefficients called loadings (*c_(r,p)_*):

$S(r)=\sum_{p} c_{(r,p)}X_{p}$ (1)

One can re-write equation (1) in matrix notation:

$S=vX$ (2)

where the elements of $v = [c_{1}\ldots c_{p}]^{T}\text{ }$(the loadings), compose the eigenvectors of the variance-covariance matrix of the original data. The eigenvalues of the variance-covariance matrix express the amount of variance explained by each principal component, with principal components typically being ordered in descending value of variance explained. Selecting the first two dominant principal components (which in our study always explained more than 40% of the variance) allows for a straightforward and quite simple interpretation of differences between any two sequences through a two dimensional plot, with the scores for each of the principal components serving as the coordinates.

# Genetic signatures of host reservoir within viral taxa

We set out to investigate whether the random forest algorithm is efficient at finding associations between differences in allele frequencies and host specificity, whilst providing a measure of variable significance. Evolutionary landscapes shape host specificity patterns at different scales. Changes that enable a given pathogen to cross the species barrier might be neutral (or even deleterious) in the “donor” host species, but are subject to strong selective pressures in the recipient host species. We would then expect the mutants under neutral selection in the donor species to have a different frequency when compared to the same mutants in the recipient hosts. Of interest is the result of selection operating within the recipient host species. Due to tissue tropism, host compatibility and transmission potential constraints, selection pressure should be extreme and the number of possible phenotypic outcomes relatively limited. Comparisons of allele frequencies between sequences of a pathogen found in humans and in rodents for instance should reveal a large subset of alleles where allele frequency does not change that much between human and rodent samples and a smaller subset of humans and rodent specific alleles. Whilst this is clear for particular pathogens or very similar strains of the same pathogen, comparisons between allele frequencies in relatively distantly related viruses might be fuzzier. As a proof of concept we implemented RFA on a set of sequences of the polymerase genes of RNA viruses to investigate the association between differences in allele frequency and host reservoir. This procedure was performed for 4 different RNA viral taxa: Flavivirus and Alphavirus geneses, and Calciviridae and Paramyxoviridae families. Table S2 lists the sequences used. Figure S3 highlights how the algorithm performs well for the Alphavirus genus but less so when dealing with more unrelated viruses, even though host reservoir clustering is observed for the Paramyxo-viruses. This is reflected in the lower robustness of the procedure for the Caliciviridae and Paramyxoviridae viruses, when compared with the Flaviviruses and Alphaviruses, and inherent difficulty in finding a highly significant and stable set of host discriminating variables (Figure S1 – right hand panels).

# Functional analysis of selected alleles

### Influenza A H1N1 HA analysis

As mentioned in the main text, this analysis serves not only as an assessment of the functional relevance of the positions being highlighted as host specific by RFA, but also as a benchmark of the algorithm through direct comparison with a recent study published by Meroz and colleagues [[38](#_ENREF_38)], in which a similar algorithm (Adaboost) is implemented. One advantage RFA has over other methods is that it is not limited to two class problems. This allows us to simultaneously compare allelic frequencies between sequences found in pre-pandemic human samples, post-pandemic human samples, and pre-pandemic swine samples. We compared the performance of RFA and Adaboost using full HA segment amino acid sequences in a significant collection of viruses (866 pre-pandemic H1N1 and 674 pandemic human H1N1 (pH1N1) viruses, together with 368 swine H1N1 viruses), mirroring that of the Meroz *et al*. study. Table S7 displays the positions identified by the RFA as significantly discriminant of the three groups analysed here.

We should also note that there is some overlap between the two sets of relevant positions presented in [[38](#_ENREF_38)]. This is a natural consequence of doing parallel analyses that can potentially pick up positions with relevance for both comparisons. Our algorithm give us the flexibility to investigate the importance of all features when particular groups are left out of the analysis and compare those importance measures with the ones obtained when using all groups. We can then identify (by *k-means* clustering) subsets of positions which are highly significant for discriminating H1N1 pandemic viruses from human and/or swine viruses (green points in Figure S5), and another subset of positions that also differentiates human from swine viruses (red points). We imposed a cluster size of 4 for the *k-means* clustering, *a priori* proposing to find a group of non-significant positions in both analyses, 2 groups of positions only significant in either one of the analyses, and a group of positions highly significant for both. The outcome demonstrates that there is no cluster of positions which are highly significant for the pre-pandemic human *vs.* swine analysis but non-significant for the complete analysis.

### Influenza A PB2 analysis

We performed feature selection on full PB2 amino acid sequences of influenza viruses encompassing 7 influenza subtypes (H1N1, H1N2, H2N2, H3N2, H5N1,H3N8, H7N7), and collected in 5 different hosts (humans, birds, pigs, dogs, and horses), as detailed in Table S8. Once the full PB2 sequences of the 7 influenza A subtypes were aligned, we undertook feature selection using RFA. A total of 48 out of 464 polymorphic alleles were selected as determinants of host species, corresponding to 23 amino acids. The host consensus amino acids in the selected positions, along with respective level of conservation for all viruses found in each of the species (δh, δa, δs, δc, δe), are presented in Table S9. Basic physiochemical properties (size, side chain charge and polarity) of the consensus amino acids are colour coded. From the table it is striking that equine and canine sequences are rather similar to each other, whereas swine sequences display the highest degree of divergence within a host. This is highlighted by Table S10 which presents the pairwise mean distances between different host reservoir groups and between sequences of the same reservoir. Swine and human sequences are significantly more divergent, displaying the most paired differences among same host reservoir sequences. Sequences collected in canine and equine hosts clearly display the most commonality, curiously followed by the avian/swine group pair. Whereas the first piece of information clearly supports the notion that phylogeny can restrict cross host transition events and evolutionary directionality; the fact that paired distances between human or swine sequences and avian sequences is smaller than between sequences found in that reference mammal host and sequences from any other mammal, hints that diversity found in swine and human samples is shaped by frequent cross host transmission of viruses from avian hosts to humans and swine hosts most likely due to the high interspecies contact rates in domestic settings.

The consensus sequence for each host was submitted to the Phyre protein structure predicting online algorithm [[39](#_ENREF_39)]. The output structures were then visualised using the VMD software [[40](#_ENREF_40)].

# References

1. Pepin KM, Lass S, Pulliam JR, Read AF, Lloyd-Smith JO (2010) Identifying genetic markers of adaptation for surveillance of viral host jumps. Nat Rev Microbiol 8: 802-813.

2. Drake JW (1993) Rates of spontaneous mutation among RNA viruses. Proc Natl Acad Sci U S A 90: 4171-4175.

3. Guyon I, Elisseeff A (2003) An Introduction to Variable and Feature Selection. Journal of Machine Learning Research 1157-1182.

4. Kimura M (1979) The neutral theory of molecular evolution. Sci Am 241: 98-100, 102, 108 passim.

5. Pybus OG, Rambaut A, Belshaw R, Freckleton RP, Drummond AJ, et al. (2007) Phylogenetic evidence for deleterious mutation load in RNA viruses and its contribution to viral evolution. Mol Biol Evol 24: 845-852.

6. Hughes AL, Hughes MAK (2007) More effective purifying selection on RNA viruses than in DNA viruses. Gene 404: 117-125.

7. Davies TJ, Pedersen AB (2008) Phylogeny and geography predict pathogen community similarity in wild primates and humans. Proc Biol Sci 275: 1695-1701.

8. Streicker DG, Turmelle AS, Vonhof MJ, Kuzmin IV, McCracken GF, et al. (2010) Host phylogeny constrains cross-species emergence and establishment of rabies virus in bats. Science 329: 676-679.

9. Pedersen AB, Altizer S, Poss M, Cunningham AA, Nunn CL (2005) Patterns of host specificity and transmission among parasites of wild primates. Int J Parasitol 35: 647-657.

10. Kuiken T, Holmes EC, McCauley J, Rimmelzwaan GF, Williams CS, et al. (2006) Host species barriers to influenza virus infections. Science 312: 394-397.

11. Li W, Zhang C, Sui J, Kuhn JH, Moore MJ, et al. (2005) Receptor and viral determinants of SARS-coronavirus adaptation to human ACE2. EMBO J 24: 1634-1643.

12. Subbarao EK, London W, Murphy BR (1993) A Single Amino-Acid in the Pb2-Gene of Influenza-a Virus Is a Determinant of Host Range. Journal of Virology 67: 1761-1764.

13. Ambroise C, McLachlan GJ (2002) Selection bias in gene extraction on the basis of microarray gene-expression data. Proc Natl Acad Sci U S A 99: 6562-6566.

14. Diaz-Uriarte R, Alvarez de Andres S (2006) Gene selection and classification of microarray data using random forest. BMC Bioinformatics 7: 3.

15. Breiman L (2001) Random forests. Machine Learning 45: 5-32.

16. Strobl C, Boulesteix AL, Zeileis A, Hothorn T (2007) Bias in random forest variable importance measures: illustrations, sources and a solution. BMC Bioinformatics 8: 25.

17. Mitchell MW (2011) Bias of the Random Forest Out-of-Bag (OOB) Error for Certain Input Parameters. Open Journal of Statistics 1: 205-211.

18. Chen CL, Andy; Breiman, Leo (2004) Using Random Forest to Learn Imbalanced Data. Statistics Technical Reports 666

19. Reif DM, Motsinger AA, McKinney BA, Crowe JE, Moore JH (2006) Feature selection using a random forests classifier for the integrated analysis of multiple data types. Proceedings of the 2006 IEEE Symposium on Computational Intelligence in Bioinformatics and Computational Biology: 171-178.

20. Lee RTC, Santos CLS, de Paiva TM, Cui L, Sirota FL, et al. (2010) All that glitters is not gold - founder effects complicate associations of flu mutations to disease severity. Virology Journal 7.

21. Parrish CR, Holmes EC, Morens DM, Park EC, Burke DS, et al. (2008) Cross-species virus transmission and the emergence of new epidemic diseases. Microbiol Mol Biol Rev 72: 457-470.

22. Zhou X, Wang X, Dougherty ER (2006) Multi-class cancer classification using multinomial probit regression with Bayesian gene selection. Syst Biol (Stevenage) 153: 70-78.

23. Leung YY, Chang CQ, Hung YS, Fung PC (2006) Gene selection for brain cancer classification. Conf Proc IEEE Eng Med Biol Soc 1: 5846-5849.

24. Fan X, Shi L, Fang H, Cheng Y, Perkins R, et al. (2010) DNA microarrays are predictive of cancer prognosis: a re-evaluation. Clin Cancer Res 16: 629-636.

25. Saeys Y, Inza I, Larranaga P (2007) A review of feature selection techniques in bioinformatics. Bioinformatics 23: 2507-2517.

26. Allen JE, Gardner SN, Vitalis EA, Slezak TR (2009) Conserved amino acid markers from past influenza pandemic strains. BMC Microbiol 9: 77.

27. Diaz-Uriarte R (2007) GeneSrF and varSelRF: a web-based tool and R package for gene selection and classification using random forest. BMC Bioinformatics 8.

28. Svetnik V, Liaw A, Tong C, Wang T (2004) Application of Breiman's random forest to modeling structure-activity relationships of pharmaceutical molecules. Multiple Classifier Systems, Proceedings 3077: 334-343.

29. Casari G, Sander C, Valencia A (1995) A method to predict functional residues in proteins. Nat Struct Biol 2: 171-178.

30. Jombart T, Devillard S, Balloux F (2010) Discriminant analysis of principal components: a new method for the analysis of genetically structured populations. BMC Genet 11: 94.

31. Paschou P, Ziv E, Burchard EG, Choudhry S, Rodriguez-Cintron W, et al. (2007) PCA-correlated SNPs for structure identification in worldwide human populations. PLoS Genet 3: 1672-1686.

32. Li JZ, Absher DM, Tang H, Southwick AM, Casto AM, et al. (2008) Worldwide human relationships inferred from genome-wide patterns of variation. Science 319: 1100-1104.

33. Novembre J, Stephens M (2008) Interpreting principal component analyses of spatial population genetic variation. Nat Genet 40: 646-649.

34. McVean G (2009) A genealogical interpretation of principal components analysis. PLoS Genet 5: e1000686.

35. Gibbs RA, Taylor JF, Van Tassell CP, Barendse W, Eversole KA, et al. (2009) Genome-wide survey of SNP variation uncovers the genetic structure of cattle breeds. Science 324: 528-532.

36. Chessel D, A.-B. Dufour, and J. Thioulouse. (2004) The ade4 package-I- One-table methods. R News 4: 5-10.

37. Jolliffe IT (2002) Principal component analysis. New York: Springer. xxix, 487 p. p.

38. Meroz D, Yoon SW, Ducatez MF, Fabrizio TP, Webby RJ, et al. (2011) Putative amino acid determinants of the emergence of the 2009 influenza A (H1N1) virus in the human population. Proc Natl Acad Sci U S A 108: 13522-13527.

39. Kelley LA, Sternberg MJE (2009) Protein structure prediction on the Web: a case study using the Phyre server. Nature Protocols 4: 363-371.

40. Humphrey W, Dalke A, Schulten K (1996) VMD: Visual molecular dynamics. Journal of Molecular Graphics & Modelling 14: 33-38.
